# Megabenthic Diversity Patterns on a Seamount in the Philippine Sea: Implications for Conservation Planning on the Kyushu‐Palau Ridge

**DOI:** 10.1002/ece3.70427

**Published:** 2024-10-17

**Authors:** Xun Lu, Chengcheng Shen, Chenghao Yang, Weikun Xu, Juan Yang, Chunsheng Wang, Dong Sun

**Affiliations:** ^1^ Key Laboratory of Marine Ecosystem Dynamics, Second Institute of Oceanography, Ministry of Natural Resources Hangzhou P. R. China; ^2^ School of Marine Science China University of Geosciences Beijing P. R. China; ^3^ National Deep Sea Center, Ministry of Natural Resources Qingdao P. R. China; ^4^ Southern Marine Science and Engineering Guangdong Laboratory (Zhuhai) Zhuhai P. R. China; ^5^ School of Oceanography Shanghai Jiao Tong University Shanghai P. R. China

**Keywords:** Beta diversity, depth gradient, environmental drivers, megafauna, seamount

## Abstract

The oligotrophic tropical western Pacific region is characterized by a high density of seamounts, with the Kyushu‐Palau Ridge (KPR) being the longest seamount chain here. Effective spatial management plans for seamount ecosystems necessitate an understanding of distribution patterns and key environmental factors influencing benthic communities. However, knowledge regarding deep‐sea biodiversity patterns over intricate topography remains limited. In this study, we investigated a seamount with a water depth of 522 m at the summit located in the southern section of KPR. Survey transects were conducted from 522 m to 4059 m. By analyzing video‐recorded data obtained by a human‐occupied vehicle (HOV) during dives and environmental variables derived from bathymetry, distinct assemblages were identified through noise clustering. α‐ and β‐diversity patterns within the seamount megabenthic community were analyzed across the depth gradient, along with investigation of their environmental drivers. A total of 10,596 megafauna individuals were documented, categorized into 88 morphospecies and statistically separated into six distinct community clusters using noise clustering analysis. Species abundance and richness were highest within the 700–800 m water depth range, declining notably beyond 2100 m, indicating a critical threshold for habitat classification in this region. The β‐diversity of megabenthic communities was high (0.836). Although β‐diversity patterns along the depth gradient were mostly dominated by differences in species richness, the contribution of species replacement increased with depth, becoming dominant at depths greater than 3000 m. Depth emerged as the primary driver of spatial variation in community structure, while near‐bottom current velocity, topographic parameters (bathymetric position index, slope), and substrate type also influenced the formation of microhabitats. The study highlights the depth gradients, thresholds, and other intricate environmental factors shaping the spatial heterogeneity of these communities. It provides valuable insights for the future development of effective survey and conservation strategies for benthic biodiversity on the KPR.

## Abstract 2

We conducted a detailed study of a seamount in the Philippine Sea and analyzed the patterns of variation and factors controlling the beta diversity of the benthic community.

## 1 Introduction

Seamounts are topographic features that rise up to 100 m above the surrounding seafloor and are important topographic features in the deep ocean (Staudigel et al. 2010). According to Clark et al. (2011), seamounts are categorized into three types based on the water depth at their summits: shallow (0–200 m), intermediate (200–800 m), and deep (> 800 m). The interaction of ocean currents with seamounts results in upwellings that transport nutrients from the deep water to the surface, facilitating high biomass and species richness, thus making seamounts important biodiversity hotspots for benthic organisms and fishes (Rowden et al. 2010; Samadi et al. 2006; Victorero et al. 2018). Due to their ecological significance, seamounts have become important areas for deep‐sea fisheries, with concerns raised about the impact of bottom trawling on the slow‐growing, long‐lived benthic communities inhabiting seamounts (Williams et al. 2010). The exploration and exploitation of deep‐sea mineral resources pose a potential threat to seamount benthos in the future (Miller et al. 2018). Effective deep‐sea spatial planning, based on habitat classification, is crucial for supporting the conservation of deep‐sea ecosystems (McQuaid, Bridges, and Howell 2023). However, the success of this approach heavily relies on understanding the distribution patterns of benthic communities and the environmental factors influencing them (McQuaid et al. 2020). Despite the importance of seamounts, ecological studies have only focused on a limited number of them, and survey effort across different seamount areas and types is uneven (Clark et al. 2012; Rogers 2018); for example, seamount summits are more commonly studied than seamount sides or bases. Additionally, while previous research has highlighted significant differences in biological assemblages on different sides of seamounts, there is a lack of repeat sampling on seamounts (Morgan et al. 2019). This uneven sampling approach may lead to a misrepresentation of the biodiversity present on seamounts.

Diversity, calculated as the number of species per sampling unit (i.e., alpha‐diversity) or through various indices (the most common being Shannon–Wiener and Margalef species richness), is a classic attribute characterizing a community. It has been consistently used in both terrestrial and marine habitats as a fundamental descriptor in ecological and conservation studies (Alsaffar et al. 2019). Beta diversity refers to the variability in species composition across different sites within a specific geographic area. It can be attributed to the rate of change or turnover in species composition along a particular environmental gradient (Legendre, Borcard, and Peres‐Neto 2005; Whittaker 1972, 1960). Considerable variation in the topography and physical environment may occur within an individual seamount which can increase the heterogeneity of the spatial structure of biological communities (Alvarez Perez et al. 2018). To better comprehend beta diversity and develop effective conservation strategies, it is beneficial to decompose beta diversity into two components: replacement and differences in species abundance or richness (Baselga 2010; Legendre 2014). The concept of replacement indicates the turnover of species along a gradient based on their ecological tolerance, while differences in richness may reflect the availability of ecological niches in different regions (Legendre 2014). There are also differences in protection strategies for the two components (Wright and Reeves 1992). The species richness difference would permit the prioritization of just a small number of the richest sites, whereas the replacement would require devoting conservation efforts to a large number of different sites, not necessarily the richest ones (Baselga 2010).

Various environmental and biological variables exhibit variability in relation to depth, making depth a crucial driver of species composition and community organization on seamounts (McClain et al. 2010). For instance, distinct depth‐related patterns are observed in benthic communities across a surveyed bathymetric range (34–1154 m) at the shallow Cobb Seamount (Du Preez, Curtis, and Clarke 2016). Moreover, depth was recognized as a significant environmental influencer of megabenthic community structure on both an immediate seamount (Lapointe et al. 2020) and a deep seamount (Shen et al. 2021). However, the impact of bathymetric gradients on alpha diversity metrics of communities, such as species richness, requires further investigation. For example, while McClain et al. (2010) did not establish a direct correlation between species diversity and depth, they did identify a beta relationship. Similarly, Bridges et al. (2022) did not find an alpha connection with depth, whereas Bridges et al. (2021) did find a beta association. Unlike alpha diversity, beta diversity shows significant variations along depth gradients on seamounts, with higher diversity observed across depth gradients compared to horizontal geographic gradients (Goode et al. 2021; McClain and Lundsten 2015; McClain and Rex 2015). Studies have highlighted that productivity influences the pattern of diversity in these diversity metrics (Stuart et al. 2017; Wagstaff et al. 2014). While research on beta diversity in seamounts has primarily focused on eutrophic areas near land, where replacement is the dominant component (Goode et al. 2021; Victorero et al. 2018), there is a lack of investigation in the extremely oligotrophic and densely distributed tropical western Pacific region. This research gap impedes our understanding of the mechanisms driving and sustaining seamount beta diversity.

Apart from depth, other environmental factors like substrate type and topography, which influence species establishment, survival, and development, are also pivotal in shaping benthic community structures on seamounts (Buhl‐Mortensen et al. 2010; Clark et al. 2010; Du Preez, Curtis, and Clarke 2016). Seamounts typically feature a high proportion of hard substrate compared to other deep‐sea habitats, leading to the prevalence of sessile epibenthic organisms on these seamounts (Rogers 2018). The irregular topographical characteristics of seamounts interact with ocean currents, affecting energy transfer, larval dispersal, colonization, and serving as key drivers of seamount benthic communities (Davies et al. 2015). Additionally, the hydrodynamic environment (Iyer et al. 2012; Jiang et al. 2021) and water masses (Henry et al. 2014; Puerta et al. 2022) also impact seamount biodiversity and community structure. For example, internal tides and local mixing on seamounts have been found to affect filter‐suspension feeders by influencing the supply and redistribution of food (Mohn et al. 2023). In addition, the hydrodynamic regime around seamounts not only affects organic matter distribution, but it has also been shown to influence the quality of organic matter (Bongiorni et al. 2013). Although research on environmental drivers of seamount benthic communities has made great progress, the primary factors that best elucidate changes in species distributions and community structures still necessitate further exploration.

In this study, we investigated an intermediate seamount with a summit of 522 m located in the southern section of the KPR in the extremely oligotrophic tropical western Pacific Ocean. We examined the species composition and community structure of megabenthic organisms, as well as the environmental drivers across the entire depth gradient from the summit edge to the base of the seamount (522–4059 m). We further explored the pattern of change in community beta diversity and its components along the depth gradient, and attempted to find the zonation boundary of the megabenthic community along the depth gradient. This threshold is critical to support habitat classification (HC) in the KPR and the Philippine Sea. Furthermore, we examined potential variations in the patterns of change along the depth gradient between communities located on different sides of a seamount. This investigation serves to inform the development of an enhanced approach for conducting deep‐sea biodiversity surveys.

## 2 Method

### 2.1 Field Site Selection

The seamount under investigation was situated to the south of the Kyushu‐Palau Ridge (KPR) in the Philippine Sea, recognized as an Ecologically or Biologically Significant Area (EBSA) under the Convention on Biological Diversity (CBD), emphasizing the need for conservation efforts in this region (CBD 2016). The topography of the southern part of the KPR is characterized by rugged terrain with uneven slopes, where the western slopes are steep and the eastern slopes are more gradual. The southern KPR features a complex topography comprising seamount groups, intramontane basins, and intermontane valleys (Qin et al. 2021). In the Philippine Basin, the North Equatorial Current (NEC) flows westward on the surface, reaching depths of around 200 m (Qiu et al. 2015). The deep‐sea circulation is governed by the Pacific Meridional Overturning Circulation (PMOC), extending from the Yap‐Mariana Junction (YMJ) to the Northern Philippine Basin (NPB) (Wang et al. 2023). The oxygen minimum zone (OMZ) occurs around 1000 m (Usui et al. 2007), leading to an oligotrophic environment with low nutrient availability, particularly nitrogen (N) and iron (Fe) (Browning et al. 2022; Yun et al. 2020; Zhang et al. 2012). Due to oligotrophy, particulate organic carbon (POC) fluxes are notably low, ranging from 0.38 to 0.43 mg m^−2^ d^−1^, representing some of the lowest carbon fluxes globally (Kempe and Knaack 1996). A recent study measured POC fluxes along the KPR at various depths and found that values decreased with depth (Wang et al. 2024). The KPR serves as a critical spawning ground for the Japanese conger (*Conger myriaster*), contributing to high fish diversity with 213 species identified, including 14 new to science (Kurogi et al. 2012; Okamura et al. 1982).

**FIGURE 1:**
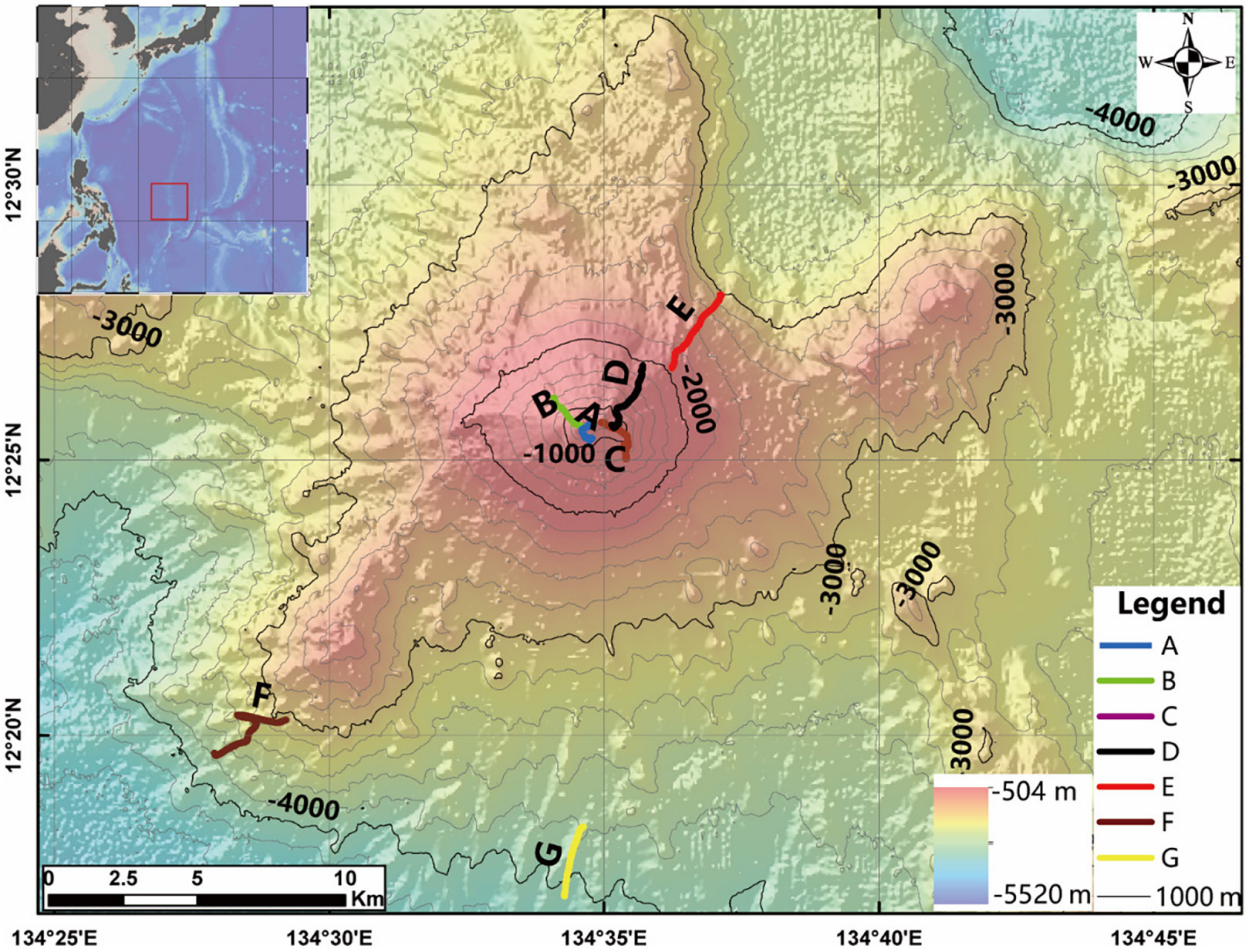
Map of the study area and survey dives, with the inset map in the upper left corner showing the location of this seamount in the Philippine Sea.

To characterize the benthic diversity of seamount groups in the southern section of KPR, we selected the shallowest seamount and designed survey lines covering the whole bathymetric range. This unnamed seamount, temporarily labeled as “KPR‐12.43” for the purpose of this study, has a summit depth of 522 m, and sides that descend to over 4000 m of water depth at the base (Figure 1).

### 2.2 Collection of Biological Data

During 2021, two cruises (DY60 and DY68) organized by the China Ocean Mineral Resources Research & Development (COMRA) were conducted to survey this seamount using the R/V *SHENHAIYIHAO* and the human‐occupied vehicle (HOV) *JIAOLONG*. This HOV is equipped with a 4‐K and 10‐fold optical zoom digital camera to collect video data, and two digital cameras (Canon PowerShot G5, 2592 × 1944 pixels) to collect HD photos.

This study assessed seven survey dives (A–E) covering a total distance of 14.2 km (Table S1). These dives spanned the entire bathymetric profile of the seamount, from its summit to its base, with depths ranging from 522 to 4059 m. Dive A focused on a water depth range of 522–860 m at the summit area. Dives B, C, and D covered depths from 532 m to 1932 m, running parallel lines on different sides of the upper slope. Dive E explored the lower slopes of the seamount at depths of 2081–2516 m, while dives F and G investigated the base at depths of 3126–4059 m.

### 2.3 Video Analysis

Based on the data obtained from the ultra‐short baseline (USBL) record of the submersible trajectory, we obtained the precise location of each dive. Consistent with prior research, the 14.2 km transects were divided into 142 samples per 100 m (Shen et al. 2021; Victorero et al. 2018). After filtering out invalid video segments (those more than 3 m above the seafloor) where organisms could not be identified, megabenthic organisms were identified to species/morphospecies; in cases where the video quality was insufficient for clear morphology distinction, higher resolution images were used to aid identification. Taxonomic classification was conducted following the World Register of Marine Species (WoRMS: http://www.marinespecies.org/), with nomenclature referenced to Horton et al. (2021). Images of ambiguous organisms were reviewed by a professional taxonomist, and those that remained unidentified were labeled as unclear fauna and excluded from statistical analysis. Due to the lack of detailed images, fishes, and crustaceans were not reliably identified as morphospecies, leading to their exclusion from the analysis. Only distinct individuals were included and listed in Table S2. Subsequently, abundance and presence‐absence data matrices of sample‐species were created for further statistical examination.

### 2.4 Collection of Environmental Data

Environmental factors encompass variables such as depth, topographic parameters (BPI, slope, roughness, curvature), substrate parameters (backscatter and substrate type as a categorical variable), and the hydrodynamic environment parameters (the mean velocity of near‐bottom current). Following the categorization principles outlined by Shen et al. (2021), substrate types were categorized into five groups based on sediment coverage: soft substrate (> 80%), moderate‐soft substrate (60%–80%), mixed substrate (40%–60%), moderate‐hard substrate (20%–40%), and hard substrate (< 20%). In cases where multiple substrates were present within a sample, the substrate type was determined by calculating the weighted average of sediment coverage. A substrate hardness score based on the types and proportions of each substrate observed on a 5‐point scale where 1 would equate to soft substrate.

Bathymetric data and backscatter data were acquired by Kongsberg EM124 sounder and processed using CARIS HIPS and SIPS 6.1 (Bongiovanni, Stewart, and Jamieson 2022; Yang et al. 2020). Gridded bathymetric data were then produced with a cell size of 100 m and used for extracting topographic variables. The topographic variables, including slope, curvature, roughness, and Bathymetric Position Index (BPI), were extracted using the Benthic Terrain Modeler (BTM) built on the ArcGIS platform (Walbridge et al. 2018). The slope is calculated from the gradient of the seabed in the direction of maximum inclination (Jerosch et al. 2016). BPI indicates the relative position of each grid cell within the overall landscape, with positive values denoting an above‐average position and negative values indicating a below‐average position. There are two scales of BPI: broad BPI and fine BPI, and the inner and outer radius for fine‐ and broad BPI were 3 and 24 and 20 and 80, respectively (De Reu et al. 2013; Fan et al. 2022; Lundblad et al. 2006). Roughness, a parameter commonly used to characterize terrain heterogeneity, was computed using the algorithm detailed in Wilson et al. (2007). The current velocity considered in this study refers to the mean velocity of near‐bottom currents, hereafter referred to as mean current velocity, with a spatial resolution of 100 m, calculated using the FVCOM model (Chen, Liu, and Beardsley 2003; Walbridge et al. 2018).

### 2.5 Statistical Analysis

Prior to analysis, species occurring only once and empty samples were excluded. In order to evaluate the relationship between pairs of environmental variables, a Pearson correlation analysis was conducted to determine the covariance among these variables, with the exclusion of those exhibiting high collinearity (Pearson *r* > 0.9). The Kruskal–Wallis test was employed to analyze the variability in the species diversity metrics of benthic community at different depths (Zar 1999). The adequacy of sampling was assessed by constructing a species accumulation curve using the *specaccum* function from the *vegan* package (Gotelli and Colwell 2001). To differentiate between megabenthic communities and identify samples with similar species composition, noise clustering analysis was carried out on normalized species data using the *vegclust* function from the *vegclust* package (Dave and Krishnapuram 1997), and a constant distance to the centroid of 0.75. The results were visualized through a non‐metric multidimensional scaling (NMDS) ordination plot. Furthermore, to evaluate the similarity in species composition among samples collected from three parallel survey dives across a depth gradient, the *metaMDS* function from the *vegan* package was utilized to conduct NMDS based on the Bray‐Curtis distance matrix (Faith, Minchin, and Belbin 1987).

In order to evaluate the spatial heterogeneity of communities across the entire seamount, the Hellinger‐transformed species abundance data were used to assess dissimilarities between samples. The total variance of the matrix was used as an estimate for beta diversity (*β*_SD_). The function *beta.div* from the *adespatial* package was employed to compute the species contribution to beta diversity (SCBD) and the sample contribution to beta diversity (LCBD) (Legendre 2014). SCBD quantifies the extent of variability among individual species, with species exhibiting higher SCBD values being more diverse within the sample and potentially serving as valuable ecological indicators. On the other hand, LCBD measures the uniqueness of the sample, where a higher LCBD value indicates a more distinct species composition within the samples (Legendre and De Caceres 2013).

In order to assess the relative importance of the two components at various depths, beta diversity (*β*_jac_) was quantified based on the Jaccard dissimilarity matrix, which was subsequently decomposed into replacement (*β*_repl_) and richness difference/abundance difference (*β*_rich_). The analysis was conducted using the *beta.div.comp* function from the *adespatial* package (Dray et al. 2012). Finally, a generalized additive model (GAM) using the *mgcv* package (Wood 2011) was utilized to examine variations in Jaccard dissimilarity and its two components across different bathymetric levels, encompassing the entire bathymetric range and three parallel survey dives.

Before distance‐based redundancy analysis (dbRDA), the environmental data was analyzed for forward selection to simplify model using the *ordistep* function (Blanchet, Legendre, and Borcard 2008). The distance matrix and environmental data were then combined in a dbRDA to explore the environmental factors influencing community structure (Legendre and Anderson 1999). Significance of the model, variables, and axes was assessed through a permutation test of the *F*‐statistic (999 permutations, *p* < 0.05) and an analysis of variance (ANOVA). Additionally, to determine the impact of individual environmental variables, hierarchical partitioning was performed using R package *rdacca.hp* (Lai et al. 2022). All the statistical analyses were conducted using the software package R v4.3.1.

## 3 Results

### 3.1 Environmental Background

**FIGURE 2:**
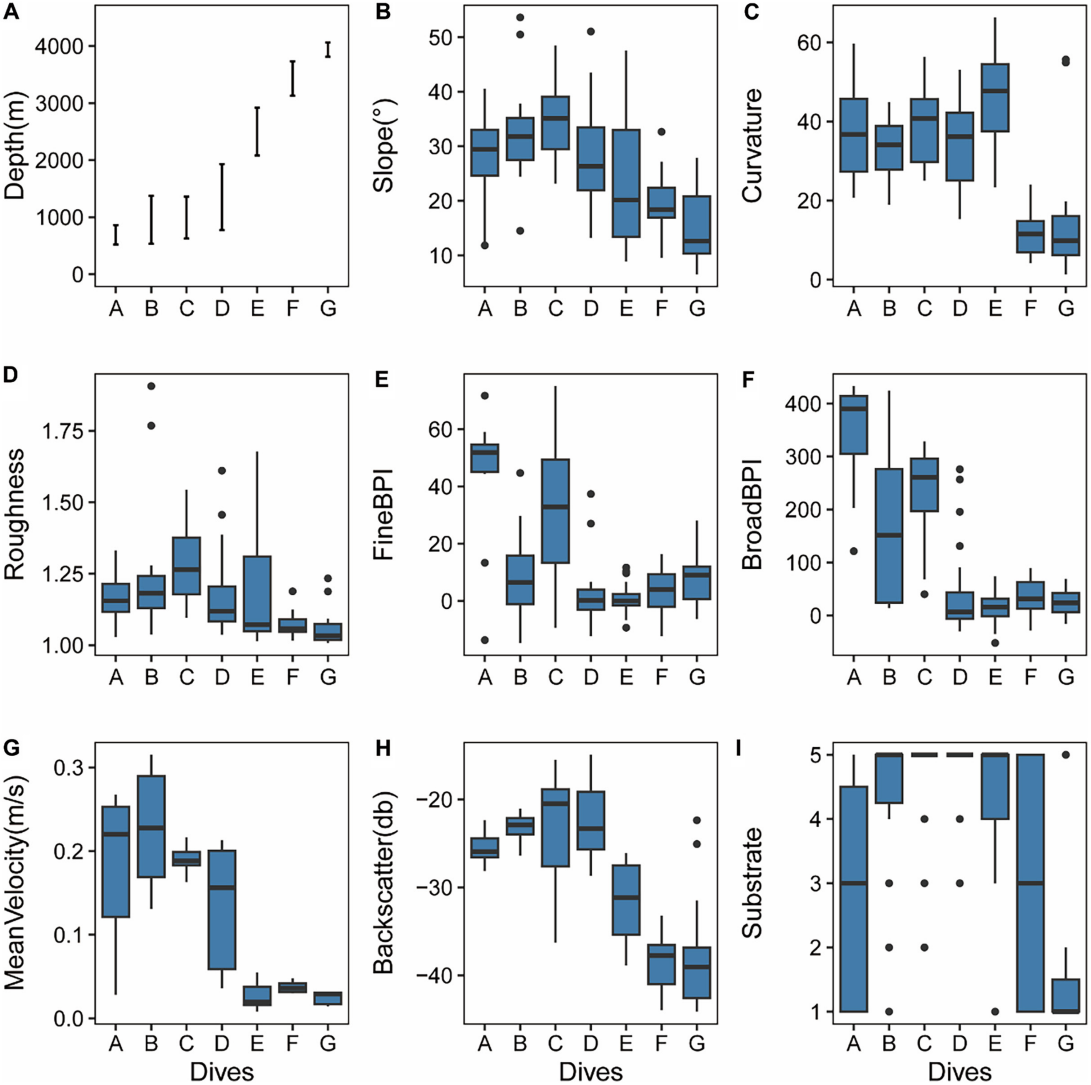
Environmental variables are presented for individual dives, organized sequentially based on increasing water depth, ranging from shallow to deep. Substrate hardness was rated for each sample using a five‐point scale, with 1: Soft substrate, 2: Moderately soft substrate, 3: Mixed substrate, 4: Moderately hard substrate, and 5: Hard substrate.

The survey line spans a total length of 14.2 km, with bathymetry coverage ranging from 520 to 4060 m, exhibiting a continuous overall bathymetry range (Figure 2). Predominant substrate types include very hard substrate at 56.3%, followed by soft substrate at 21.1%, and mixed substrate at 12.7%. Pearson correlation analysis revealed significant (*p* < 0.01) negative correlations between depth and mean current velocity (−0.79), broad BPI (−0.645), fine BPI (−0.422), slope (−0.502), backscatter (−0.83), and substrate type (−0.408). There was no significant correlation found with aspect (*p* > 0.05). Mean current velocity showed significant (*p* < 0.01) negative correlations with backscatter, slope, broad BPI, and fine BPI, with *r* = 0.655, 0.592, 0.597, and 0.316, respectively. Backscatter and substrate type were significantly (*p* < 0.01) and positively correlated, *r* = 0.497 (Figure S1).

### 3.2 The Species Composition and Community Structure of Benthic Megafauna

In this study, a total of 10,596 megafauna individuals were documented, categorized into 88 morphospecies and 358 unclear individuals (Table S2). Among the unclear individuals, there were 19 sponges, 40 corals, 13 actiniarians, 9 crinoids, 4 holothurians, 21 asteroids, 96 ophiuroids, 6 echinoids, 3 pennatulids, and 147 individuals that could not be assigned to a phylum. Sixteen morphospecies were identified as occurring only once within the taxa. Out of the 142 sample areas examined, megafauna was absent in 9 samples, and only one morphospecies was observed in 12 samples. The abundance of five morphospecies showed high dominance: *Primnoidae* spp. 1 (sparse) indet. contributed 22.4% of the total abundance, followed by *Primnoidae* spp. 2 (dense) indet. (17.9%), *Paracalyptrophora* spp. indet. (9.1%), *Plexauridae* spp. indet. (5.7%), and *Pheronema carpenteri* sp. inc. (5.5%). These five morphospecies accounted for 60.6% of the total individual.

**FIGURE 3:**
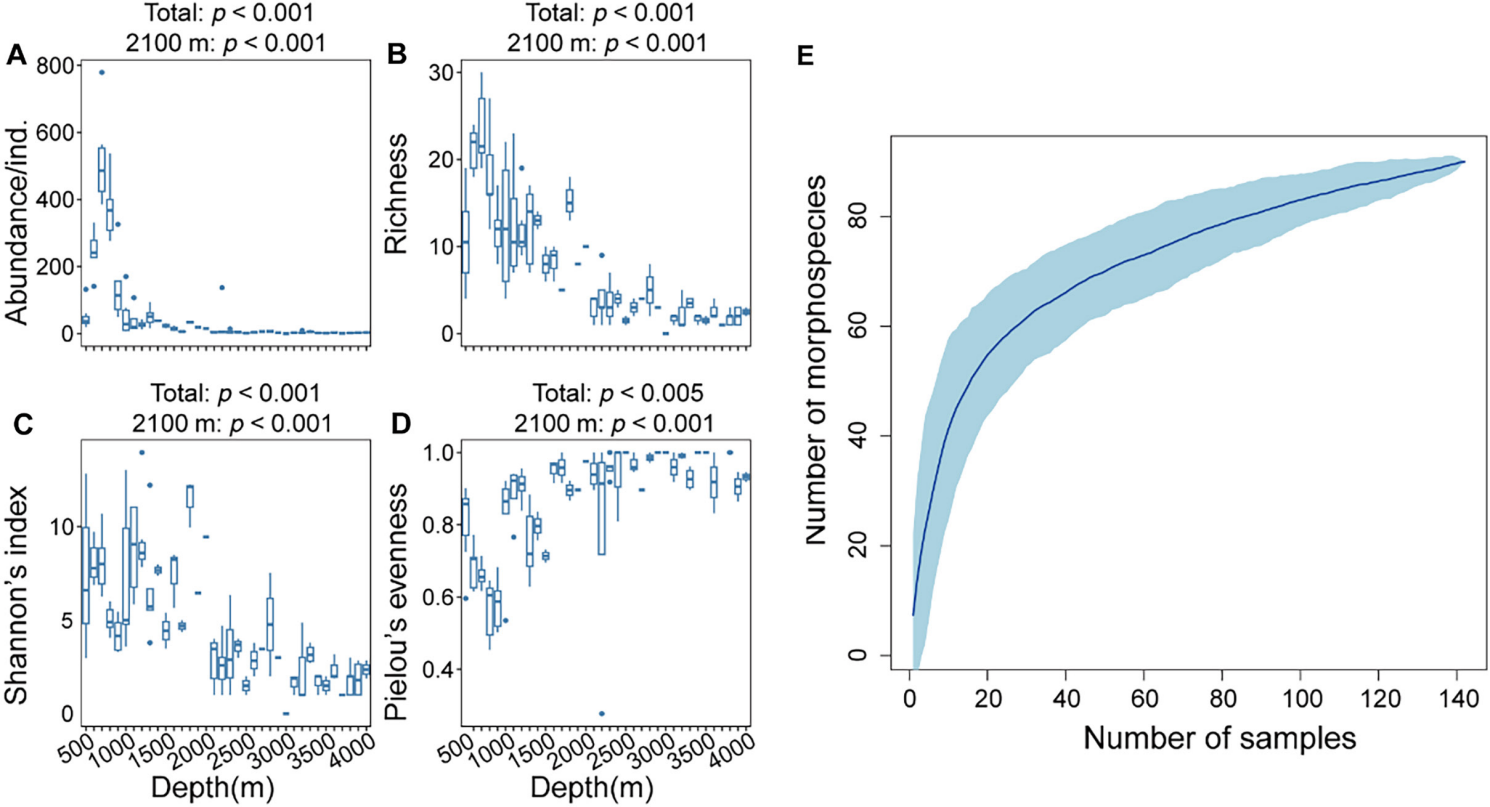
Species abundance and diversity metrics across the depth gradient binned into 100 m intervals ((A) Abundance, (B) Species richness, (C) Shannon diversity index, (D) Pielou evenness index). The total *p*‐value refers to the significance of the difference in each index across the depth gradient, while the second *p*‐value is the significance of the difference in each index between the samples before and after 2100 m. (E) Species accumulation curve where the shaded area denotes the 95% confidence intervals.

The Kruskal–Wallis test revealed significant differences in diversity metrics among megabenthic communities at various depths. Seamounts exhibited an average abundance and richness of 78.63 ± 149.38 ind./sample, and 8.52 ± 7.43 morphospecies/sample, respectively. The highest species abundance and richness were observed at depths ranging from 700 to 800 m, with mean values of 511 ± 126.46 ind./sample and 23.13 ± 4.09 morphospecies/sample, respectively. The peak values were recorded at 700 m, with 779 individuals and 30 species. Conversely, the Wilcoxon test indicated a significant decrease in abundance and richness beyond depths of 2100 m, with mean values of 5.81 ± 16.93 ind./sample and 2.75 ± 1.89 morphospecies/sample, respectively (Figure 3). Furthermore, species abundance and richness were found to be higher on hard substrates compared to soft substrates. Abundance and richness on the hardest substrate were 94.81 ± 148.55 ind./sample and 10.22 ± 6.80 morphospecies/sample, respectively, while on the softest substrate, they were 9.30 ± 15.40 ind./sample and 3.39 ± 3.80 morphospecies/sample. The species accumulation curve did not reach an asymptote, indicating the need for additional sampling to comprehensively evaluate the diversity of megafauna (Ugland, Gray, and Ellingsen 2003).

**FIGURE 4:**
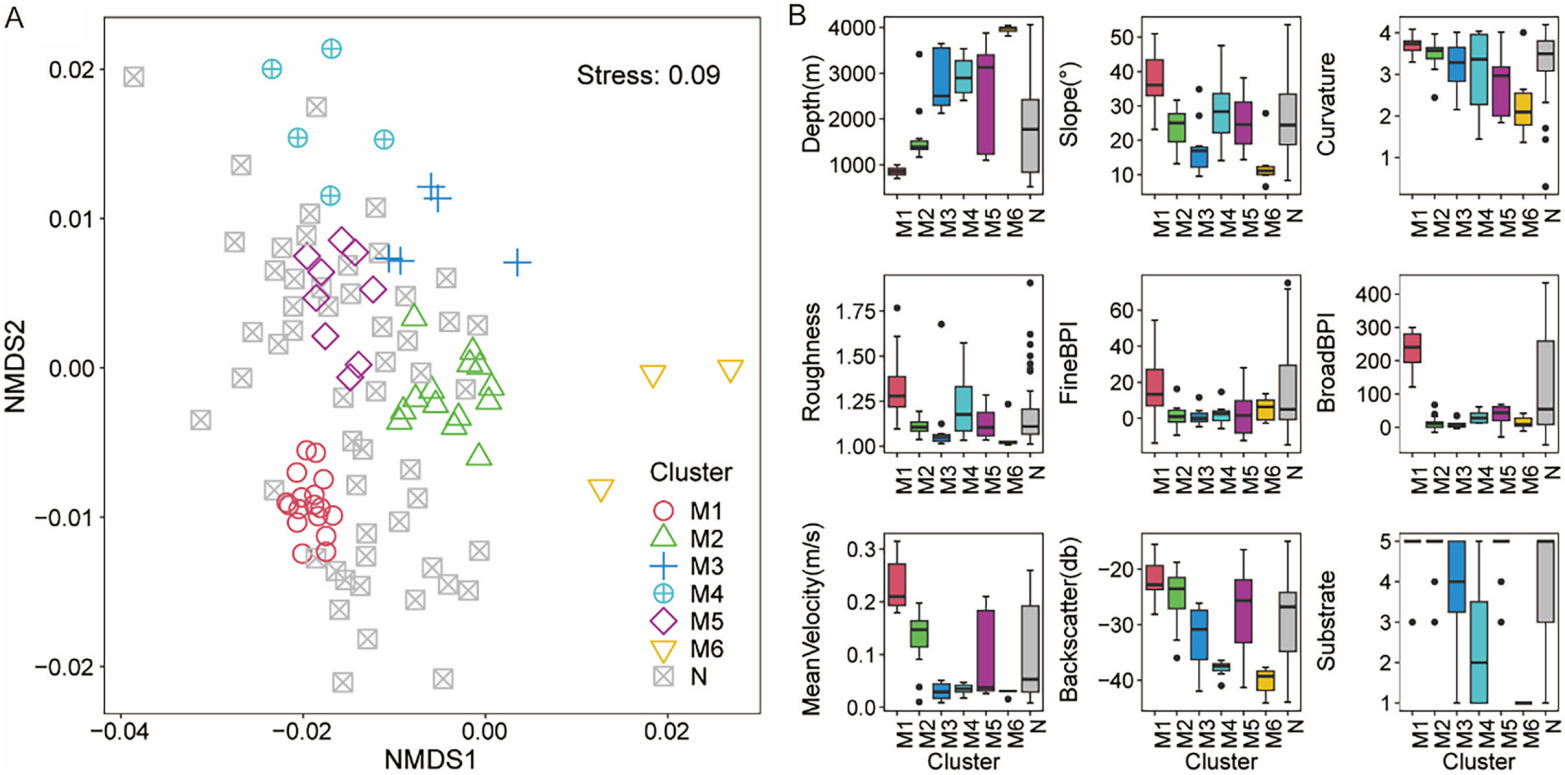
(A) Noise clustering ordination plot to identify samples with similar species composition, with gray indicating noise points (samples that cannot be grouped according to the constant distance to the centroid); (B) Box plots of environmental variables for each cluster.

**FIGURE 5:**
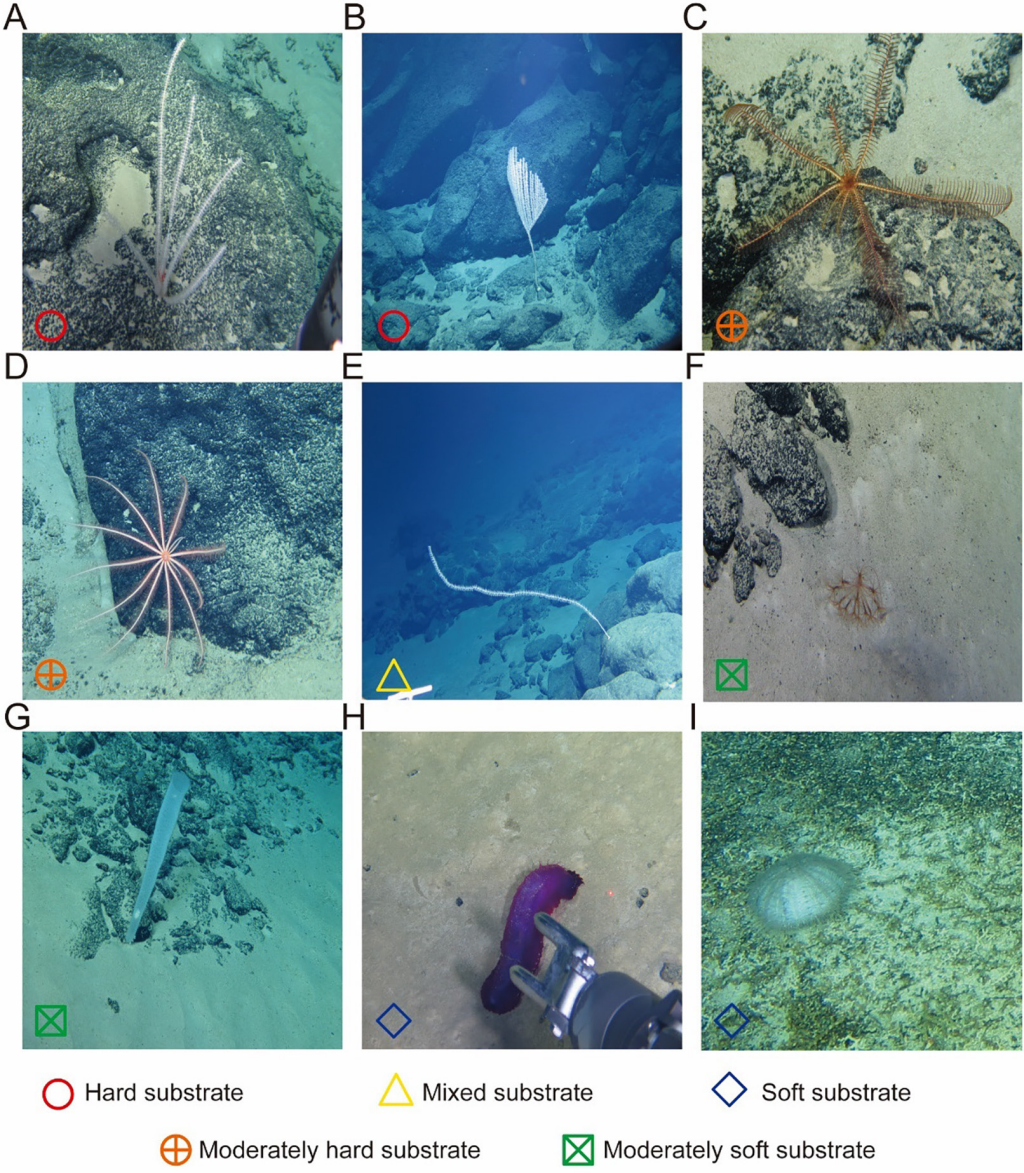
Examples of the typical species and their corresponding habitats. The species names from (A) to (I) are *Primnoidae* spp. 1 (sparse) indet., *Primnoidae* spp. 2 (dense) indet., *Pentametrocrinidae* spp. indet., *Brisinga* spp. indet., *Lepidisis* spp. indet., *Umbellula* spp. indet., *Dictyaulus* spp. indet., *Benthodytes* spp. indet., and *Echinothuriidae* spp. indet. The shapes in the bottom left corner of each picture represent the type of substrate in which the organism is found.

The noise clustering analysis resulted in the identification of six distinct clusters, encompassing 52.46% of all samples (Figure 4A). Cluster M1 comprises samples collected from dive A at the summit of the seamount, as well as dives B, C, and D along the slopes, at water depths ranging from 700 to 1000 m (Figure 4B). This cluster is characterized by a hard substrate, high mean current velocity, elevated broad BPI, and high backscatter levels. The biological composition of these samples predominantly includes species such as *Primnoidae* spp. indet., *Paracalyptrophora* spp. indet., and *Plexauridae* spp. indet (Table S3). Cluster M2 primarily consists of samples obtained at water depths between 1167 and 1570 m from dive B and dive D, featuring hard substrate and organisms identified as *Pentametrocrinidae* spp. indet., accompanied by *Strotometra* spp. indet. and *Lepidisis* spp. indet. Cluster M3 encompasses samples from dive E on the lower side and dive F at the base, at water depths ranging from 2127 to 3645 m, characterized by low mean current velocity and slope, with predominant species being *Brisinga* spp. indet. Cluster M4 includes samples from dive E on the lower slope and dive F at the base, at water depths between 2600 and 3536 m, where the substrate is soft, and the main organisms identified are *Benthodytes* spp. indet. Cluster M5 is distinguished by a predominantly hard substrate, with species such as *Lepidisis* spp. indet. and *Caulophacus* spp. indet. Cluster M6 comprises samples from dives G, featuring low mean current velocity and soft substrate, with organisms mainly identified as *Umbellula* spp. indet. (Figure 5).

### 3.3 Beta Diversity

**FIGURE 6:**
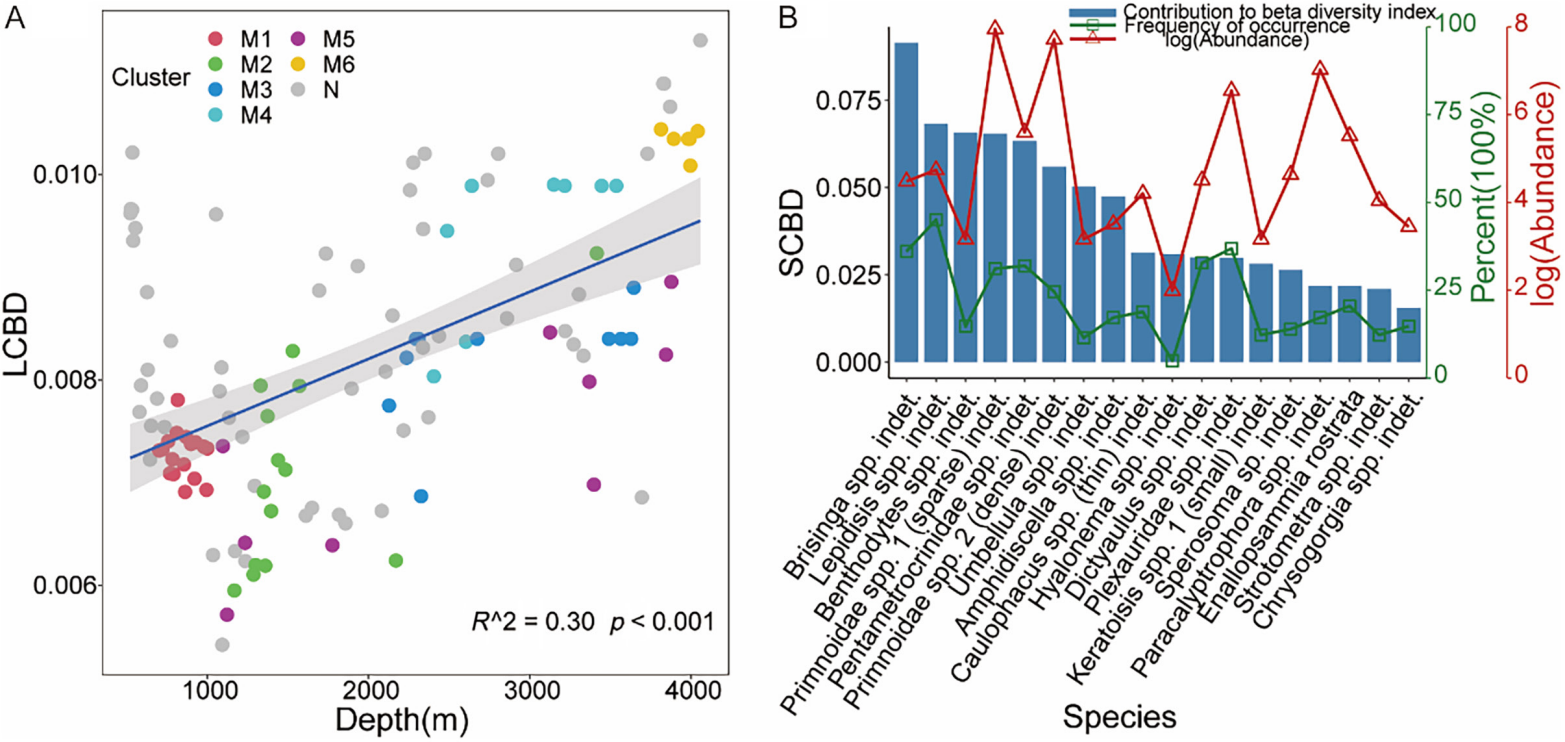
(A) Contribution of samples to beta diversity for each taxonomic cluster; (B) The 18 species with the highest contribution to beta diversity and their abundance and frequency of occurrence.

The beta diversity, as determined by the total variance, was 0.836, indicating a notable level of heterogeneity within the megafauna community. The influence of individual samples on beta diversity tended to rise with increasing depth, with cluster M6 exhibiting a higher LCBD compared to clusters M1 and M2 (Figure 6A). *Brisinga* spp. indet. and *Lepidisis* spp. indet. with high frequency of occurrence contribute greatly to beta diversity. The contribution of the most abundant *Primnoidae* taxa was also high, mainly at water depths of 700–1000 m. *Strotometra* spp. indet., which occurred in small‐scale aggregations, contributed relatively less to beta diversity (Figure 6).

Utilizing abundance data to partition the beta diversity into replacement and abundance difference components revealed that species abundance difference was the predominant driver of variation, explaining 71.73%. Similarly, when using presence‐absence data, species richness difference explained 55.73% of the variation, with species replacement contributing 44.27%. Differences between summit and base regions were evident: species abundance or richness differences were dominant at the summit, while species replacement played a larger role at the base. Similar patterns were observed across three dives on the slopes, with species abundance difference predominating in abundance data and species replacement in presence‐absence data (Table 1).

**TABLE 1:** Proportion (%) of the contribution of replacement and abundance differences/richness differences to beta diversity across the bathymetric gradient and at different locations on seamounts, based on the Jaccard dissimilarity matrix calculated from (A) abundance data, (B) presence‐absence data.

Dives	Total	Dive A	Dive B	Dive C	Dive D	Dive E	Dive F	Dive G
Position		Summit	Upper slope			Lower slope	Base	
(a) Species abundance data								
Replacement	28.27	30.76	32.95	33.51	41.62	47.02	**50.98**	**67.64**
Abundance difference	**71.73**	**69.24**	**67.05**	**66.49**	**58.38**	**52.98**	49.02	32.36
(b) Species presence‐absence data								
Replacement	44.27	43.74	**65.39**	**57.49**	**59.38**	**52.57**	**60.97**	**77.34**
Richness difference	**55.73**	**56.26**	34.61	42.51	40.62	47.43	39.03	22.66

*Note:* Bold values indicates the larger of the two parameters, replacement and richness/abundance differences.

**FIGURE 7:**
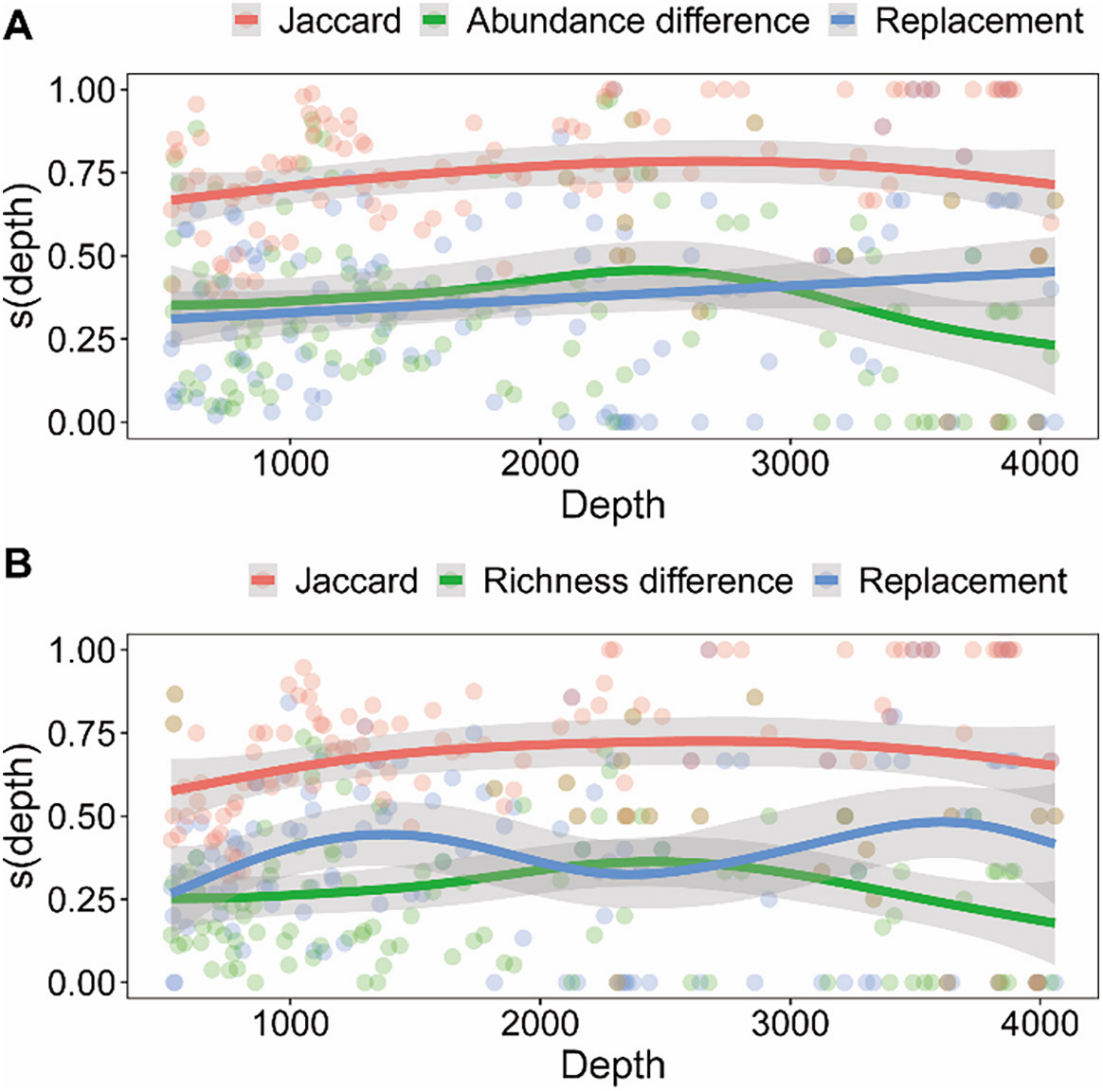
Total beta diversity and two components change with depth were analyzed using (A) abundance data, (B) presence‐absence data.

The beta diversity, as measured by Jaccard dissimilarity utilizing both abundance and presence‐absence data, exhibited similar trends along the depth gradient. Samples within the 2000–3000 m depth range displayed the greatest beta diversity. Nevertheless, the proportions of species replacement and abundance (richness) difference contributing to beta diversity varied with depth. Species abundance difference was more dominant at depths shallower than 3000 m, whereas species replacement became increasingly predominant at depths exceeding 3000 m (Figure 7A). The contribution of species replacement exceeds the richness difference in most depth ranges, except in the 2000–3000 m depth range where the former contributes less than the latter (Figure 7B).

**FIGURE 8:**
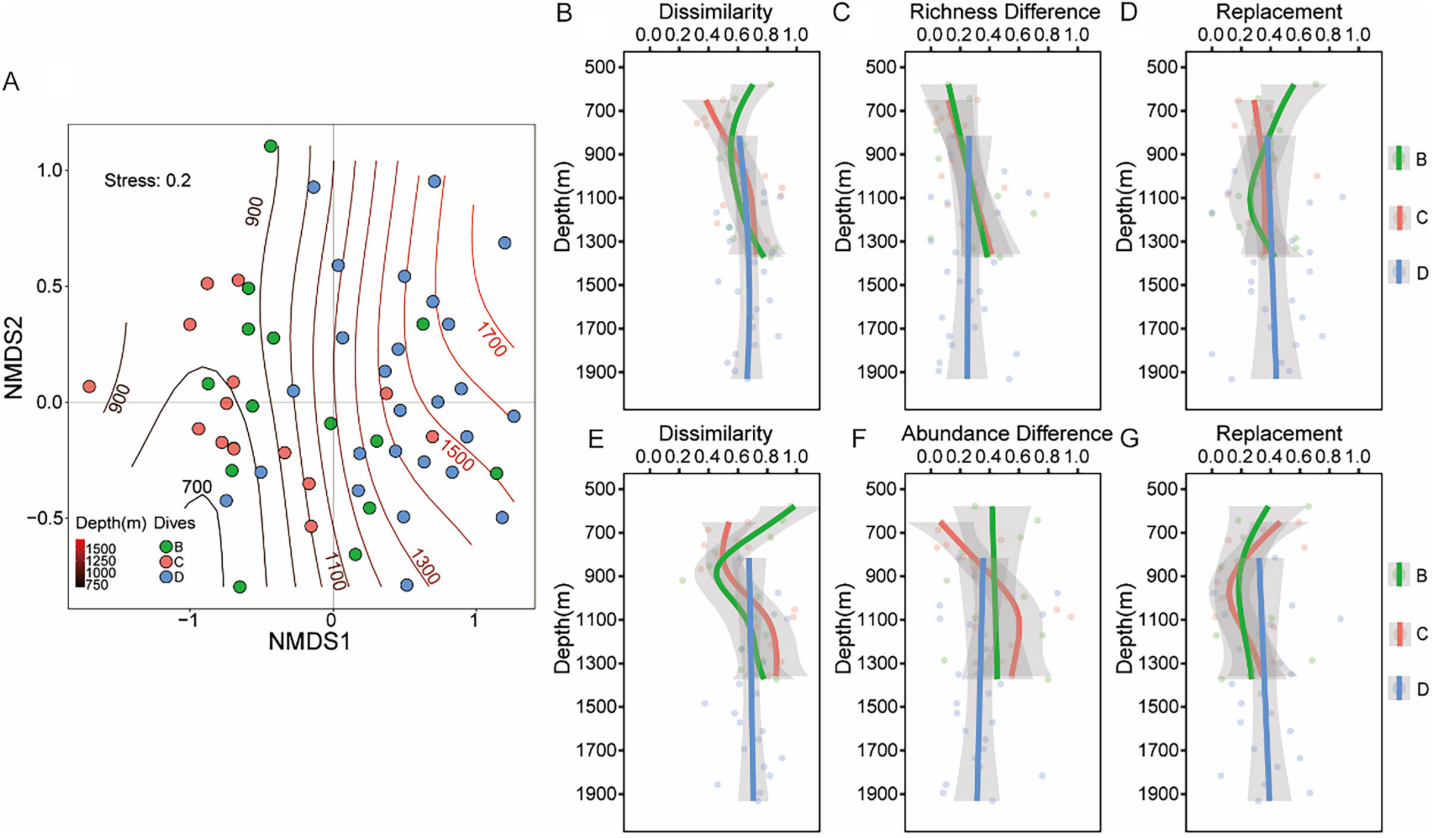
(A) NMDS showing community structure versus depth for three parallel survey dives; scatters indicate the values of the two NMDS values for the community structure of samples on the hillside, with the three survey dives indicated by different colors. Lines indicate isobaths, and numbers next to line segments indicate depth, which increases from right to left. (B) GAMs to distinguish the difference of beta diversity pattern among three parallel survey lines along the depth gradient, indicated by different colors for three parallel survey lines. (A–D) using species presence‐absence data, (E–G) using species abundance data.

The NMDS analysis revealed no statistically significant differences in species composition among the three parallel dives (Dive B, C, and D) across the depth gradient (Figure 8A). However, there was a variation in beta diversity along the depth gradients between dives conducted on different sides (Figure 8B). In the presence‐absence data, the patterns of total beta diversity and species replacement along the depth gradient diverged for dives B and C, with dive C showing a peak around 1100 m and dive B exhibiting a minimum around 1000 m. Conversely, in the abundance data, the beta diversity trend for dive B mirrored that of the presence‐absence data, indicating a minimum, while dive C displayed more pronounced fluctuations in beta diversity (Figure 8E). The total beta diversity and its components did not display distinct depth‐related patterns for dive D (Figure 8A,B).

### 3.4 Environmental Drivers

The primary environmental factors that made notable contributions to beta diversity encompassed mean current velocity, depth, substrate type, BPI, slope, and curvature. Notably, mean current velocity emerged as the most influential factor in shaping total beta diversity. Depth exhibited the greatest impact on both abundance and richness differences, with BPI following closely behind. Conversely, no environmental variables were found to be significantly linked to species replacement (Table 2).

**TABLE 2:** Effects of environmental variables on community change based on results from distance‐based redundancy analysis (dbRDA).

	$R_{\mathrm{adj}}^{2}$	*F* value	Mean current velocity	Depth	Substrate	Broad BPI	Fine BPI	Curvature	Slope	Aspect	Backscatter	Roughness
(a) Abundance data												
Bray–Curtis dissimilarity(log)	0.1563	3.0377	18.93%**	15.02%**	14.45%**	17.22%**	11.14%**	3.15%**	6.62%**	1.99%	8.68%*	2.90%
Jaccard dissimilarity	0.1480	2.9106	19.60%**	14.74%**	12.80%**	17.54%**	11.26%**	3.71%**	6.46%**	2.17%	8.29%*	3.49%
Abundance difference	0.1122	4.8223	21.34%**	30.72%**	5.73%	10.87%	8.08%**	2.91%*	4.33%	1.09%	11.67%	3.20%
Species replacement			8.41%	6.26%	27.13%	14.20%	10.61%	7.33%	8.32%	5.54%	6.30%	5.87%
(b) Present‐absence data												
Bray–Curtis dissimilarity	0.1404	2.7971	17.46%**	14.89%**	16.41%**	16.61%**	11.13%*	3.05%*	6.33%**	2.26%	9.29%*	2.60%
Jaccard dissimilarity	0.1933	3.8997	18.07%**	15.23%**	15.43%**	17.16%**	11.69%**	2.80%**	6.26%**	2.10%	9.05%	2.39%
Richness difference	0.0718	3.3386	22.54%**	30.32%**	11.14%	7.22%*	4.84%**	2.96%	4.65%	0.50%	13.28%	2.59%
Species replacement	0.0005	1.0588	7.67%	8.33%	24.37%	18.24%	11.84%	5.50%	7.99%	4.99%	6.23%	4.82%

*Note:* Environmental variables were used as explanatory variables, while (a) species abundance data (b) species presence‐absence data were used as response variables. $R_{\mathrm{adj}}^{2}$ represents the adjusted *R* ^2^, *F* value is pseudo *F*‐Statistics, and percent denotes hierarchical partitioning identified for each environmental variable for the proportion of relative importance of changes in community structure. Asterisks represent that the results of forward selection based on distance redundancy analysis (dbRDA) showed a significant effect of the environmental variable on each parameter, ** indicates *p* < 0.01 and * indicates *p* < 0.05.

**FIGURE 9:**
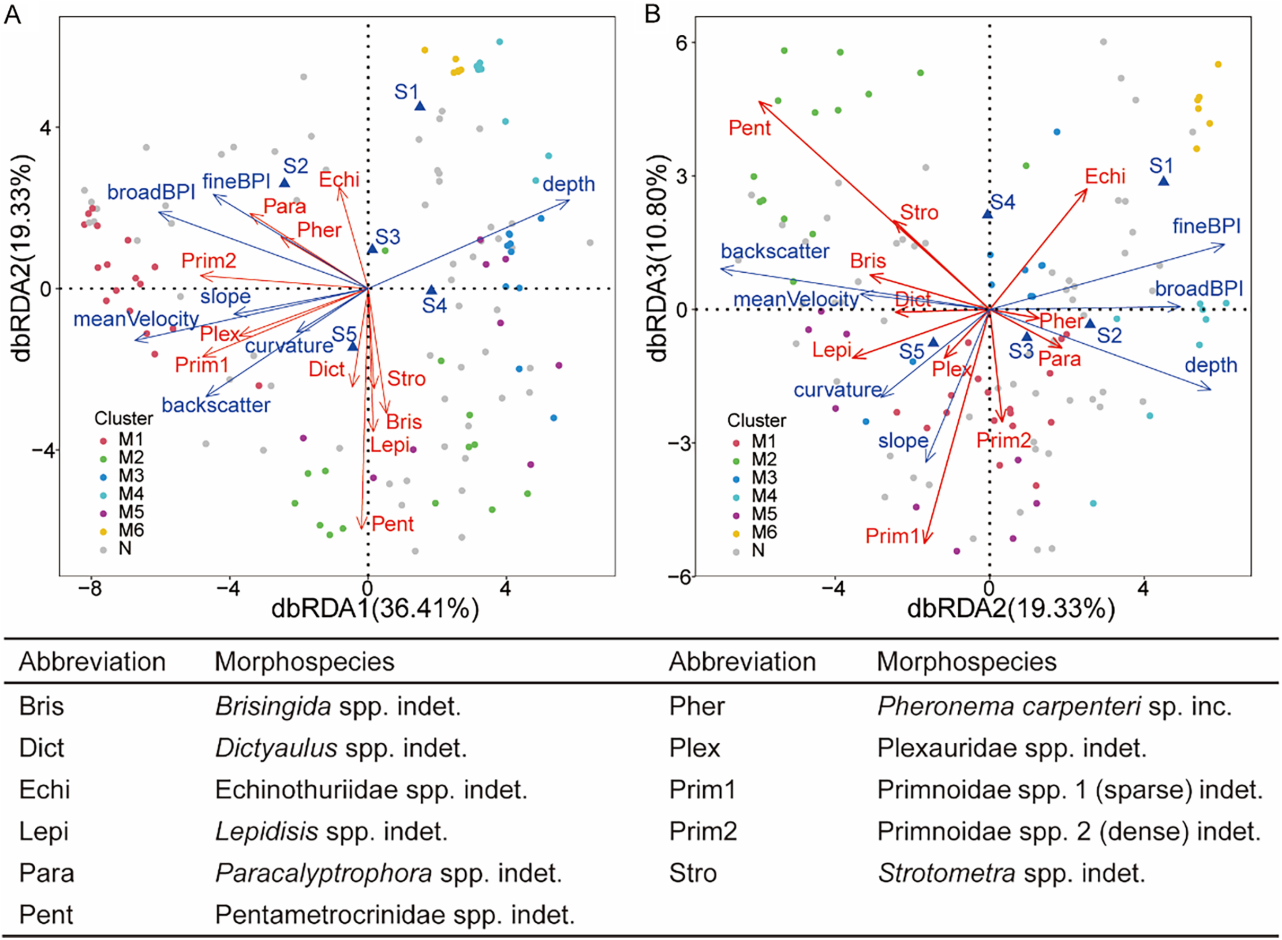
The plot of the distance‐based redundancy analysis (dbRDA) with forward selection to investigate the environmental drivers that impact community structure (A) the first and second axes (B) the second and third axes as the abscissa and ordinate using Bray–Curtis dissimilarity matrix based on species abundance data. Scattered dots indicate sample areas within the study area, and clusters are represented by different colors. Blue arrows indicate environmental variables, and discrete substrate types are shown by triangles labeled with S1: Soft substrate, S2: Moderately soft substrate, S3: Mixed substrate, S4: Moderately hard substrate, and S5: Hard substrate. Red arrows indicate species; only specific representative species were selected and represented by abbreviations. The table displays the morphospecies that correspond to the abbreviations.

The forward selection method identified seven environmental factors that showed significant associations with community structure (*p* < 0.05). These factors comprised mean current velocity, broad BPI, depth, curvature, fine BPI, substrate, slope, and backscatter. The first two axes (dbRDA1 and dbRDA2) of dbRDA accounted for 36.41% and 19.33% of the variability in species composition, respectively (Figure 9). Mean current velocity, depth, and backscatter played important explanatory roles in the first ordination axis (dbRDA1). Additionally, slope and curvature were found to covary well with the first axis. Both fine BPI and broad BPI played an important role in both the first and second ordination axes. Soft and moderate‐soft substrate types were positively correlated with ordination scores for samples distributed along the second ordination axis, while hard substrates displayed negative correlations with samples positioned along the second axis.

The results of the distance‐based redundancy analysis (dbRDA) indicated that the various species displayed distinct environmental preferences. For example, the *Primnoidae* spp. indet. and *Plexauridae* spp. indet. showed a preference for shallow water depths, whereas *Pentametrocrinidae* spp. indet., *Strotometra* spp. indet., and *Lepidisis* spp. indet. exhibited a preference for hard substrates. The *Pentametrocrinidae* spp. indet. contributed significantly to the second axis and correlated with *Lepidisis* spp. indet., *Strotometra* spp. indet., *Brisinga* spp. indet., and *Dictyaulus* spp. indet., all of which favored hard substrates (Figure 9A). M4 and M6 were soft substrate, deep‐water environments in the dbRDA1 and dbRDA2. Additionally, the separation of samples in M4 and M6 was better delineated by dbRDA2 and dbRDA3, with cluster M6, represented by *Echinothuriidae* spp. indet., indicating a preference for softer substrates compared to M4 (Figure 9B).

## 4 Discussion

### 4.1 Megabenthic Assemblages on the Seamount

In this study, species abundance and richness were found to be relatively high across the study area on the KPR‐12.43 seamount, with notable peaks in alpha‐diversity at the summit edge, particularly between 700 and 800 m. The predominant organisms identified on the seamount were filter feeders, such as corals and sponges, with habitat‐forming species like *Dendrophylliidae* spp. (huge) indet. distributed in patches on the seamount. These species contributed to habitat heterogeneity, altered ecological niches, and provided habitats for various organisms, including Ophiuroidea, shrimps, and *Paguroidea* spp. indet. (Bo et al. 2012; Buhl‐Mortensen et al. 2010; Du Preez, Curtis, and Clarke 2016). These findings align with previous studies that have highlighted the importance of biogenic habitats in maintaining high biodiversity levels and functional redundancy, as well as their resilience to environmental changes and lower susceptibility to species loss (de la Torriente et al. 2020). Compared with the Weijia seamount, also located in the oligotrophic western Pacific, the KPR‐12.43 seamount hosts a higher diversity of megabenthic organisms (88 morphospecies along a 14.2 km line compared to the 76 morphospecies along a 24.4 km line reported in Shen et al. (2021)). Weijia seamount is a deep‐water, guyot seamount with an average summit water depth of 1600 m, much deeper than the 522 m summit depth of KPR‐12.43 seamount (Clark et al. 2011).

The organisms within the research area can be broadly categorized into six distinct assemblages based on dominant species, distribution range, and environmental preferences. Among these groupings, taxa from three clusters exhibit depth preferences suggesting some level of depth stratification: (1) *Primnoidae* spp. indet., *Plexauridae* spp. indet., and *Paracalyptrophora* spp. indet. that were primarily located on the upper slopes at depths shallower than 1000 m; (2) *Pentametrocrinidae* spp. indet and *Strotometra* spp. indet. found between 1000 and 2000 m; and (3) *Umbellula* spp. indet., and *Echinothuriidae* spp. indet. found below 4000 m. Filter feeders, such as corals and sponges, are primarily found in depths shallower than 2000 m. In deeper water environments, organisms are predominantly comprised of *Umbellula* spp. indet. and *Echinothuriidae* spp. indet.

### 4.2 Environmental Drivers of Community Structure Along Depth Gradient

This study emphasized depth as a critical environmental factor influencing changes in megabenthos species composition and community structure. This is consistent with the findings of many previous studies (Bridges et al. 2021; Eerkes‐Medrano et al. 2020; Goode et al. 2021). Depth in marine ecosystems is a multifaceted representation of various environmental variables such as temperature, oxygen, and salinity (Puerta et al. 2020). The physiological tolerance of deep‐sea organisms to the physical and chemical characteristics of water masses plays a crucial role in shaping biodiversity patterns along depth gradients (Tittensor et al. 2010); similarly, temperature is an important variable driving biodiversity in marine ecosystems (Yasuhara and Danovaro 2016). Minor temperature variations of 1°C–2°C along depth gradients have been observed to impact the distribution of coral and sponge species (Dijkstra et al. 2021). A previous research on oligotrophic shallow seamount revealed a notable increase in species richness with depth, peaking at 650 m, which closely correlates with a water temperature of 5°C–6°C at that depth (Morgan et al. 2019). In the present study, the temperature at the depth of maximum species richness was approximately 5.9°C (Figure S2). Consequently, in oligotrophic seamounts, a peak in species richness at 700 m may be linked to a prevailing temperature of around 6°C. Additionally, the availability of food sinking from the surface is crucial for the survival of benthic organisms (Rogers 2018). A recent study examining the flux of particulate organic carbon (POC) across the KPR depth gradient indicated a decline in POC flux with increasing depth (Wang et al. 2024). Furthermore, POC flux was found to be positively associated with seafloor biomass and abundance (Wei et al. 2010), suggesting that diminished nutrient availability also contributes to lower species abundance and richness in samples deeper than 2100 m.

The mean current velocity, topographic factors (BPI, slope) and substrate type emerged as important drivers influencing the community composition in the research. Seamounts interacting with oceanic currents generate complex physical processes that enhance the complexity of the hydrodynamic environment, impacting sediment deposition and subsequently determining the substrate type (Boehlert and Genin 1987; Cao et al. 2022; Jiang et al. 2021). Seamount summits typically exhibit flat topography where slower currents facilitate accumulation, resulting in a softer substrate predominantly inhabited by sea urchins and sea stars. Species abundance peaks were observed at a depth of 700 m, situated at the edge of the summit where the topography is rugged. Accepted to substrate type, in oligotrophic waters, nutrients transported by water currents also play a vital role for these organisms (Boehlert and Genin 1987; Clark et al. 2010). For instance, research has shown that seamount interact with ocean currents to elevate local flow velocities, promoting the resuspension of near‐bottom layers (Jiang et al. 2021). The research indicated that the abundance and diversity of megabenthos were notably low on the lower slopes and bases at depths exceeding 2000 m. This can be attributed to various factors, including the diminished mean current velocity, uniform depositional conditions, and limited availability of food particles in this region. Collectively, these factors constrain the proliferation of filter‐feeding organisms reliant on hard substrates (Beaulieu 2001; Carney 2005).

This study did not find any environmental variables significantly associated with species replacement, suggesting the variables here did not adequately represent the biologically‐relevant variables. We may have overlooked other crucial environmental and spatial predictors, as well as variables linked to factors like biological interactions and stochastic events (Chase 2010; Devercelli et al. 2016).

### 4.3 Beta Diversity Along the Depth Gradient

In this study, high beta diversity was observed on the seamount and the contribution of samples to beta diversity generally increased with depth. It should be noted that the taxonomic identities of some organisms were not precisely resolved to the species or genus level, which could potentially influence the calculation of beta diversity. High LCBD values may suggest sites with unique species compositions and high conservation significance, or sites that are degraded and have low species richness (Legendre 2014). For instance, in this study, cluster M6 displayed high LCBD values due to its samples from depths exceeding 3800 m and lower species abundance, making them distinct from other samples. A study on fish also identified a negative correlation between high LCBD values of communities and species richness (López‐Delgado, Winemiller, and Villa‐Navarro 2020). Therefore, caution is advised when utilizing LCBD for conservation purposes, particularly for samples with unique species compositions but low species richness. Moreover, previous research has shown a positive correlation between beta diversity and substrate diversity (Hanafi‐Portier et al. 2024; Shen et al. 2021), which may explain the higher LCBD observed in clusters M3 and M4, encompassing various substrate types. Species with high SCBD found in this study were patchy in distribution and usually had high abundance in a limited depth range, such as *Primnoidae* spp. 1 (sparse) indet. and *Primnoidae* spp. 2 (dense) indet. The aggregation of species with larger SCBDs in a limited number of samples is a phenomenon also observed in other seamounts (Hanafi‐Portier et al. 2024; Victorero et al. 2018).

Beta diversity was found to exhibit an increasing trend with depth at shallower than 3000 m, followed by a decrease between 3000 m and 4000 m. Previous study proposed a positive correlation between beta diversity and environmental heterogeneity (Ellingsen and Gray 2002). This relationship likely accounts for the high beta diversity values observed within the depth range of 2000–3000 m, which approximately corresponds to the boundary between the transitional water layer and North Pacific Deep Water (Figure S2). Variations in water mass characteristics, such as temperature, salinity, density, and oxygen levels, play a significant role in shaping benthic fauna distribution patterns at different depths (Corre et al. 2022; Rogers 2018; Victorero et al. 2018). Oceanographic variability and more available ecological niches resulting from the interaction between different water masses lead to increased beta diversity, which also explains that the beta diversity reached high values within 2000–3000 m.

The importance of two components of β diversity changes with depth, where species richness difference is predominant at the summit, while species replacement dominates at the base region (Table 1), reflecting different ecological processes influencing beta diversity. Species replacement occurs when species are substituted along gradients due to environmental sorting, spatial constraints, or historical factors (Qian, Ricklefs, and White 2005). The unique environmental conditions at seamount bases, characterized by low temperature and food availability, act as environmental filters shaping the megabenthic community structure (Wang et al. 2024). In contrast, the dominance of species richness difference suggested that the relative contribution of species loss or gain on the observed pattern is more significant than species turnover (Carvalho, Cardoso, and Gomes 2012). Selective extinction, competition, colonization, and dispersal limitation all have the potential to result in species loss or gain (Brown and Cahill Jr 2022; Carvalho, Cardoso, and Gomes 2012). On the summit and its edge areas, the dominant species assemblage was M1 cluster, typified by corals and sponges with weak connectivity, and increased species abundance and richness (Figure 3), so it is possible that species gain plays a significant role in controlling beta diversity in this area. In addition, the variation in the relative contributions of the two components of beta diversity in different areas provides valuable insights for developing conservation strategies on this seamount. The dominance of species richness difference underscores the importance of prioritizing conservation efforts in areas shallower than 2100 m, exhibiting high levels of species abundance and richness. However, the phenomenon of species replacement dominating beta diversity at the base also suggests the need to devote conservation efforts to a large number of different sites. Hence, it is imperative to consider the community composition and ecological dynamics in various geographical areas when devising conservation plans, taking into account the spatial variability of habitats on the seamount.

### 4.4 Differences in Beta Diversity Patterns on Different Sides of Seamount

The three parallel survey lines exhibited similarities in community composition, but variations in beta diversity were observed along the depth gradient of all three dives (Figure 7). More unstalked crinoids, such as *Pentametrocrinidae* spp. indet. and *Strotometra* spp. indet., were discovered in the Dive B compared to the Dive C, which had a higher abundance and broader distribution of sessile fauna, such as corals and sponges. These unstalked crinoids were observed to move quickly, possibly suggesting a higher dispersal potential than other taxa (Shen et al. 2021; Williams et al. 2010). The study proposes that high dispersal rates may interfere with species sorting and thus reduce species replacement, which may explain the reduced beta diversity of Dive B (Ferreira et al. 2019; López‐Delgado, Winemiller, and Villa‐Navarro 2020). The variations in the mean current velocity and BPI among the three sides (Figure 1) may also explain the differences in their beta diversity patterns. This is because the mean current velocity and BPI are crucial factors that influence the variability among communities (Table 2). Additionally, bottom currents in opposite directions affect different sides of seamounts, further influencing ecological processes (Wang et al. 2023; Yoshioka, Endoh, and Ishizaki 1988).

The variations observed among the three facets further illustrate the heterogeneity of seamount habitats, where a range of ecological processes can coexist even within comparable bathymetric zones of seamounts. Morgan et al. (2019) undertook a replicated sampling approach across three sides of a seamount to compare the diversity and structure of assemblages with varying depths. Their results revealed high beta diversity (0.81–0.88) between the three sides, indicating that distinct microhabitats on the seamount foster unique assemblages on each side. Several investigations have documented and quantified the presence of patchy habitats on seamounts (Goode et al. 2021; Swanborn et al. 2023). The habitat heterogeneity both among and within seamounts offers suitable environments for diverse organisms, thereby influencing species diversity and distribution, ecological niche partitioning, and patterns of beta diversity (Anderson, Tolimieri, and Millar 2013; Clark et al. 2012).

In summary, species dispersal rates, the hydrodynamic environment, and microhabitat changes on different sides of seamounts combine to cause differences in beta diversity patterns, and therefore more precise quantification of spatial patterns of seamount biodiversity is needed. In future seamount surveys, repeated sampling of the bathymetric section on different sides of seamounts is needed to obtain a more comprehensive understanding of seamounts.

## 5 Conclusion

This study evidences the high biodiversity found in benthic communities located on a seamount within the oligotrophic tropical western Pacific region, particularly near the summit edge, and at depths lower than 2100 m. Depth, representing variables such as temperature, food availability, and water mass, critically influenced observed variations in alpha and beta diversity on the seamount. Variations in beta diversity patterns between locations on seamount highlight the importance of accounting for spatial heterogeneity in benthic community composition when formulating management strategies or designating protected areas. Additionally, the differences in beta diversity patterns across the three sides suggest that the high biodiversity and complexity of seamount ecosystems make it difficult for single sampling to fully capture their real diversity. This study provides a reference for the conservation and management of data‐poor KPR seamounts.

## Author Contributions

**Xun Lu:** data curation (equal), formal analysis (equal), methodology (equal), writing – original draft (equal). **Chengcheng Shen:** data curation (equal), funding acquisition (equal), investigation (equal), methodology (equal), writing – original draft (equal). **Chenghao Yang:** data curation (equal), investigation (equal). **Weikun Xu:** data curation (equal), investigation (equal). **Juan Yang:** supervision (equal). **Chunsheng Wang:** supervision (equal). **Dong Sun:** conceptualization (lead), funding acquisition (equal), project administration (equal), writing – original draft (equal), writing – review and editing (equal).

## Conflicts of Interest

The authors declare no conflicts of interest.

## Supporting information


Appendix S1.

## Data Availability

All data and R code files are available at the following link: https://zenodo.org/records/10889308.
