# Supplementary material for: Megabenthic Diversity Patterns on a Seamount in the Philippine Sea: Implications for Conservation Planning on the Kyushu‐Palau Ridge
Source: Ecol Evol. 2024 Oct 17;14(10):e70427. doi: 10.1002/ece3.70427 (PMC11486664; doi:10.1002/ece3.70427)

# Supplementary materials

## Supplementary Table S1.

Information on the HOV dives used in this study, summarizing the dives, their depths, their location, number of dives, and the beginning and end points of each dive (beginning longitude–end longitude; beginning latitude–end latitude).

| Cruise | Dives | Location | Data (yyyy/mm/dd) | Depth/m | Longitude/◦E | Latitude/◦N |
| --- | --- | --- | --- | --- | --- | --- |
| DY60 | JL169 | upper slope, NE | 2021/1/7 | 630-1355 | 134.5827-134.5899 | 12.4177-12.4280 |
| DY60 | JL175 | upper slope, SW | 2021/1/18 | 544-1366 | 134.5683-134.5771 | 12.4267-12.43464 |
| DY60 | JL177 | summit | 2021/1/22 | 529-852 | 134.5792-134.5796 | 12.4233-12.4272 |
| DY60 | JL178 | base, SW | 2021/1/23 | 3812-4058 | 134.5713-134.5759 | 12.2857-12.3044 |
| DY68 | JL199 | base, W | 2021/11/16 | 3126-3731 | 134.4655-134.4837 | 12.3253-12.3399 |
| DY68 | JL201 | upper slope, NE | 2021/11/19 | 746-1960 | 134.5863-134.5964 | 12.4375-12.4460 |
| DY68 | JL202 | lower slope, NE | 2021/11/20 | 2082-2941 | 134.6037-134.6188 | 12.4497-12.4662 |

## Supplementary Table S2.

Taxonomic list and example images of the megabenthic species or morphospecies distinguished on this seamount. It contains 86 species/morphospecies and four species of fishs and two species of shrimps. Taxonomic nomenclature follows the recommendations of Horton et al. (2021). The open taxonomic nomenclature signs used are as follows: indet.: indeterminabilis, inc.: incerta. When an organism cannot be identified exactly to the species level and is observed only once, the singular (sp.) is used. The spp. is used to indicate the presence of more than one species.

| Taxonomy | Names of species/morphospecies | Example images |
| --- | --- | --- |
| **PHYLUM: CNIDARIA** |  |  |
| Class: Anthozoa  (Subclass: Octocorallia) |  |  |
| Order: Scleralcyonacea |  |  |
| Family: Chrysogorgiidae | *Chrysogorgia* spp. indet. | 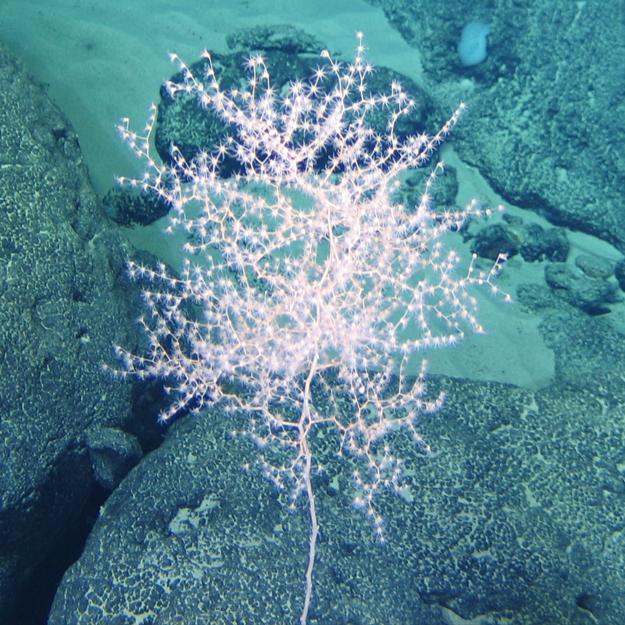 |
|  | *Metallogorgia* spp. indet. | 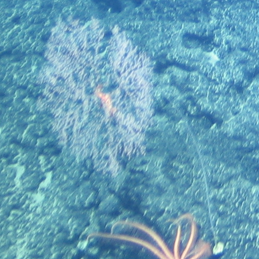 |
|  | *Iridogorgia* spp. indet. | 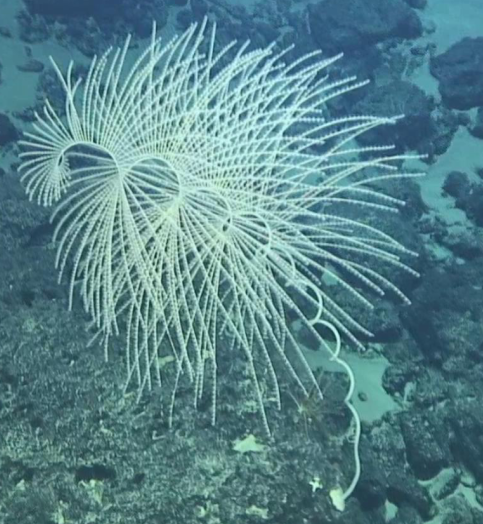 |
|  | *Ramuligorgia militaris* | 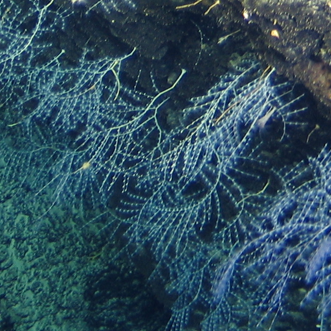 |
|  | *Rhodaniridogorgia* spp. indet. | 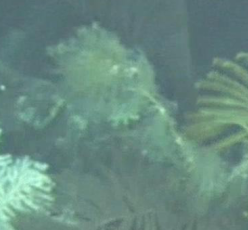 |
| Family: Primnoidae | Primnoidae spp. 1 (sparse) indet. | 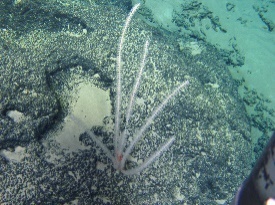 |
|  | Primnoidae spp. 2 (dense) indet. | 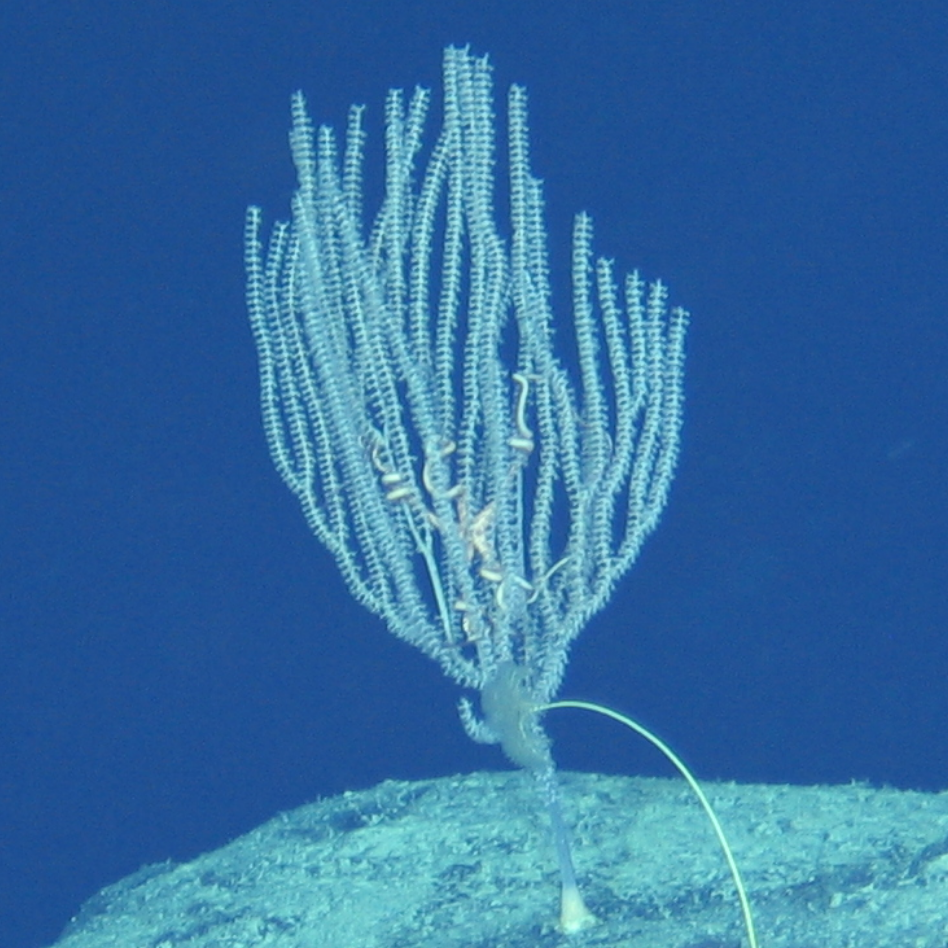 |
|  | Primnoidae spp. 3 (harp-shaped) indet. | 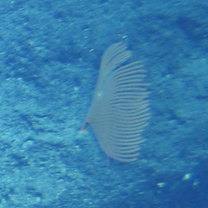 |
|  | *Callogorgia* spp. indet. | 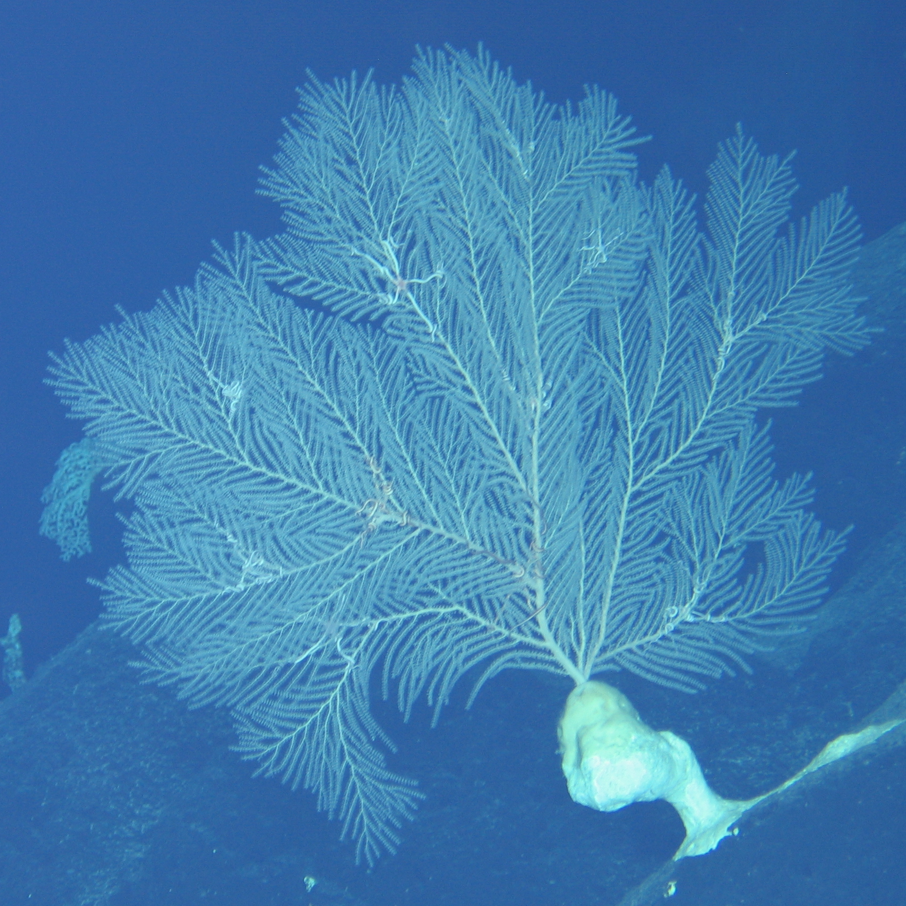 |
|  | *Paracalyptrophora* spp. indet. | 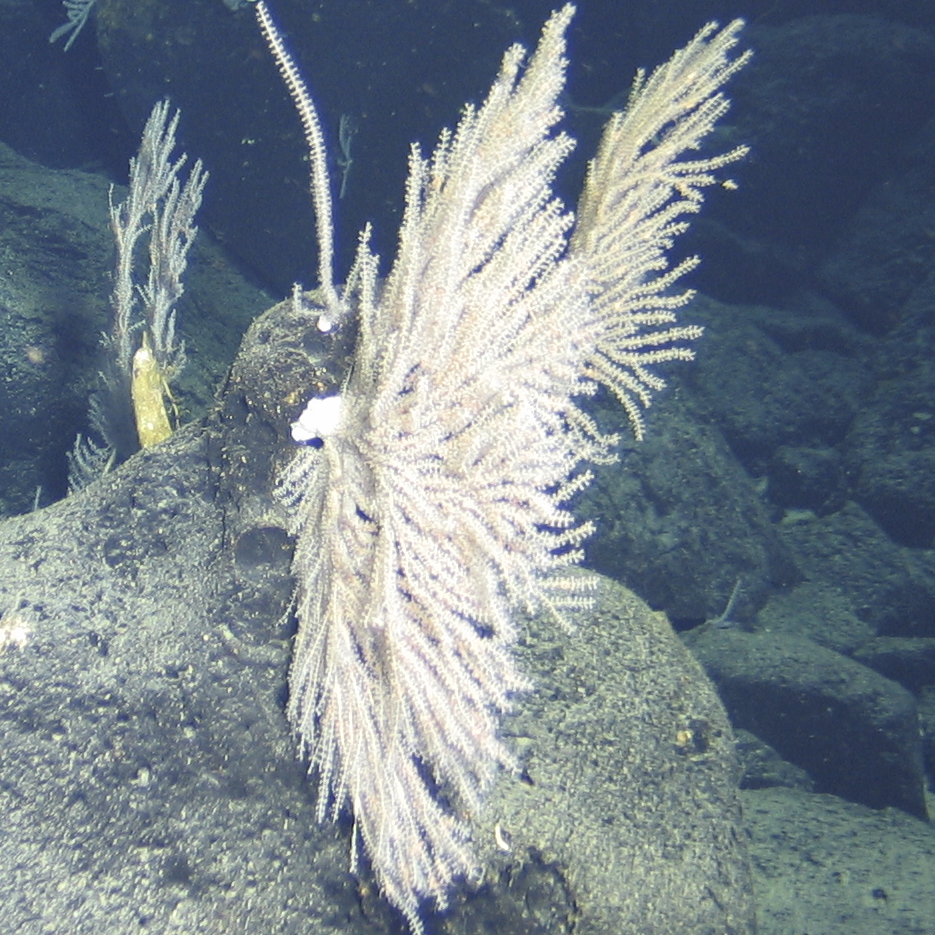 |
| Family: Keratoisididae  (Subfamily: Keratoisidinae) | *Lepidisis* spp. indet. | 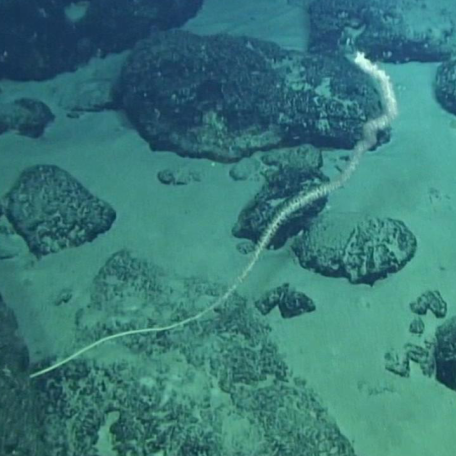 |
|  | *Keratoisis* spp. 1 (sparse) indet. | 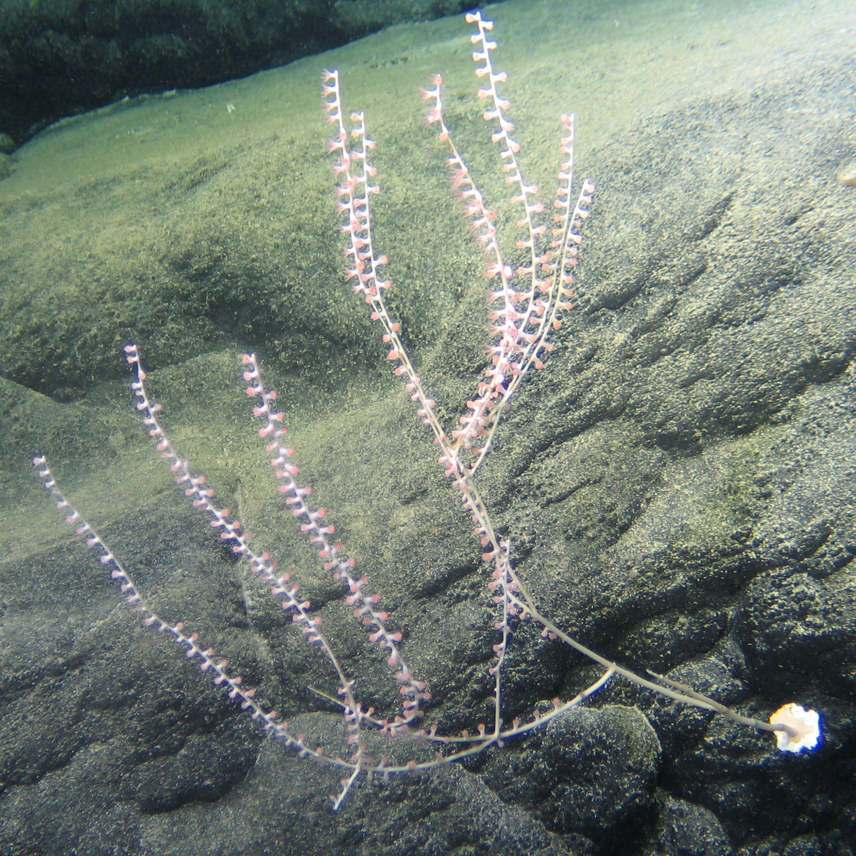 |
|  | *Keratoisis* spp. 2 (dense) indet. | 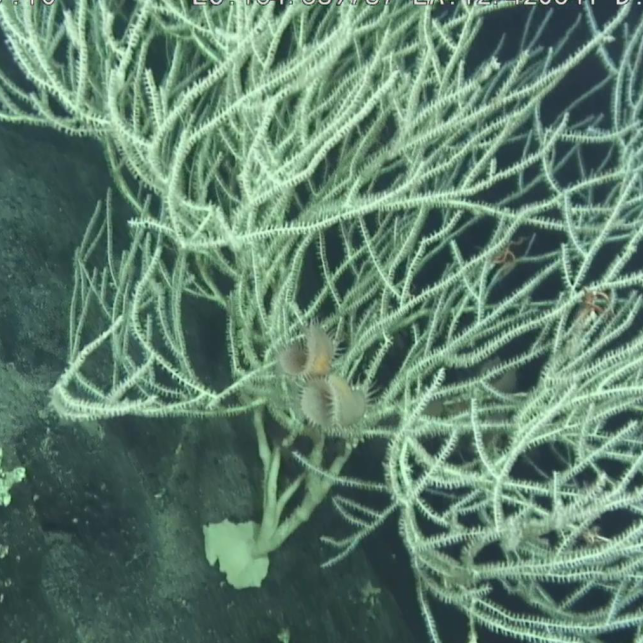 |
|  | *Isidella* spp. indet. | 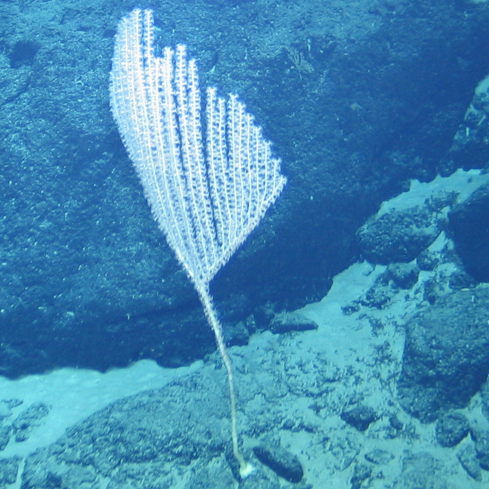 |
| Family: Coralliidae | *Paragorgia* spp. indet. | 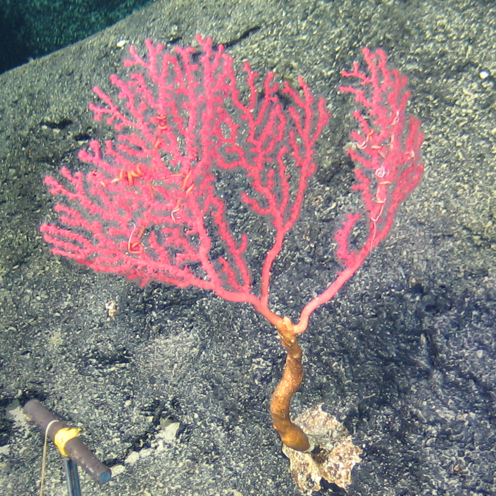 |
|  | *Hemicorallium* spp. indet. | 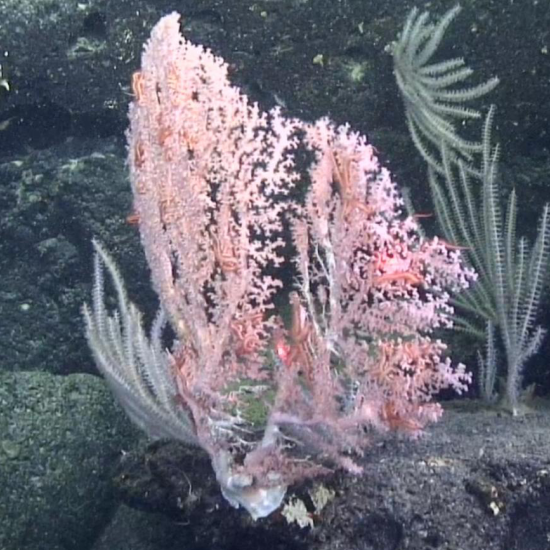 |
| (Subfamily: Anthomastinae) | *Anthomastus* spp. indet. | 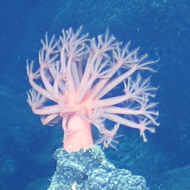 |
| Superfamily: Pennatuloidea  Family: Umbellulidae | *Umbellula* spp. indet. | 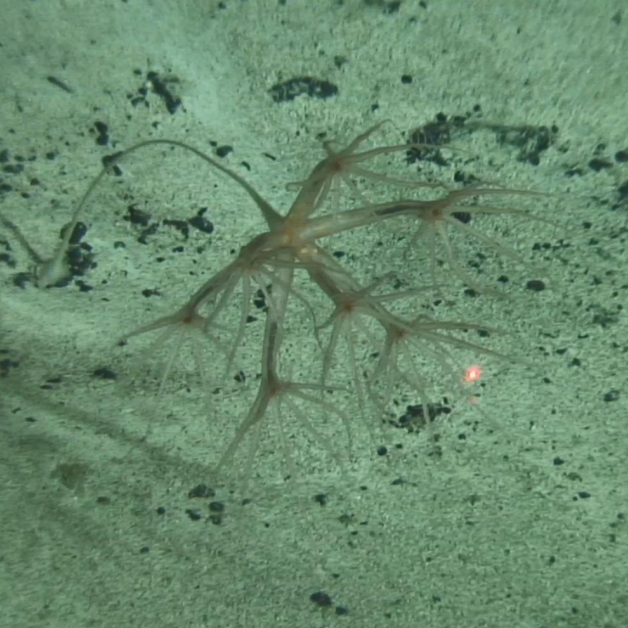 |
| Family: Halipteridae | Halipteridae sp. indet. | 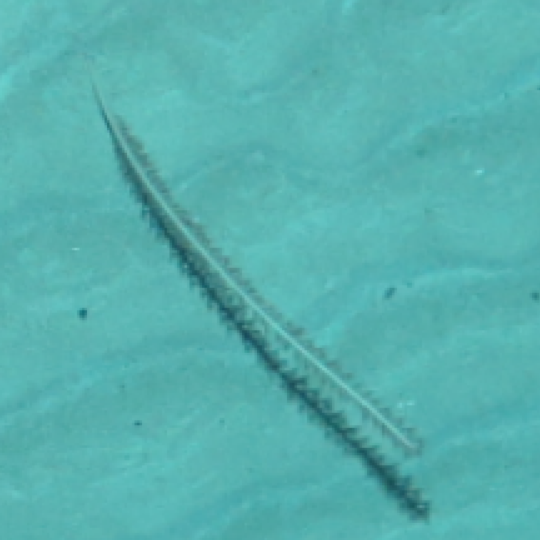 |
| Order: Malacalcyonacea |  |  |
| Family: Victorgorgiidae | *Victorgorgia* spp. indet. | 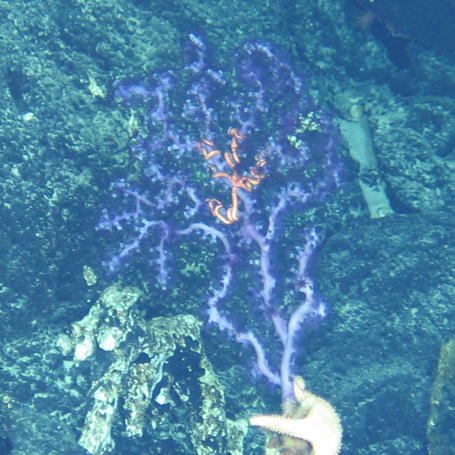 |
| Family: Plexauridae | Plexauridae spp. indet. | 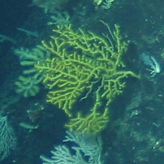 |
| Family: Paramuriceidae | *Acanthogorgia* spp. indet. | 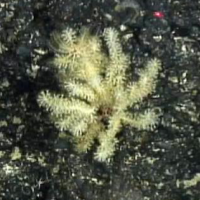 |
| (Subclass: Hexacorallia) |  |  |
| Order: Scleractinia |  |  |
| Family: Dendrophylliidae | *Enallopsammia rostrata* sp. inc. | 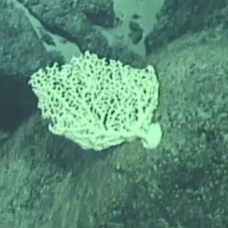 |
|  | Dendrophylliidae spp. (huge) indet. | 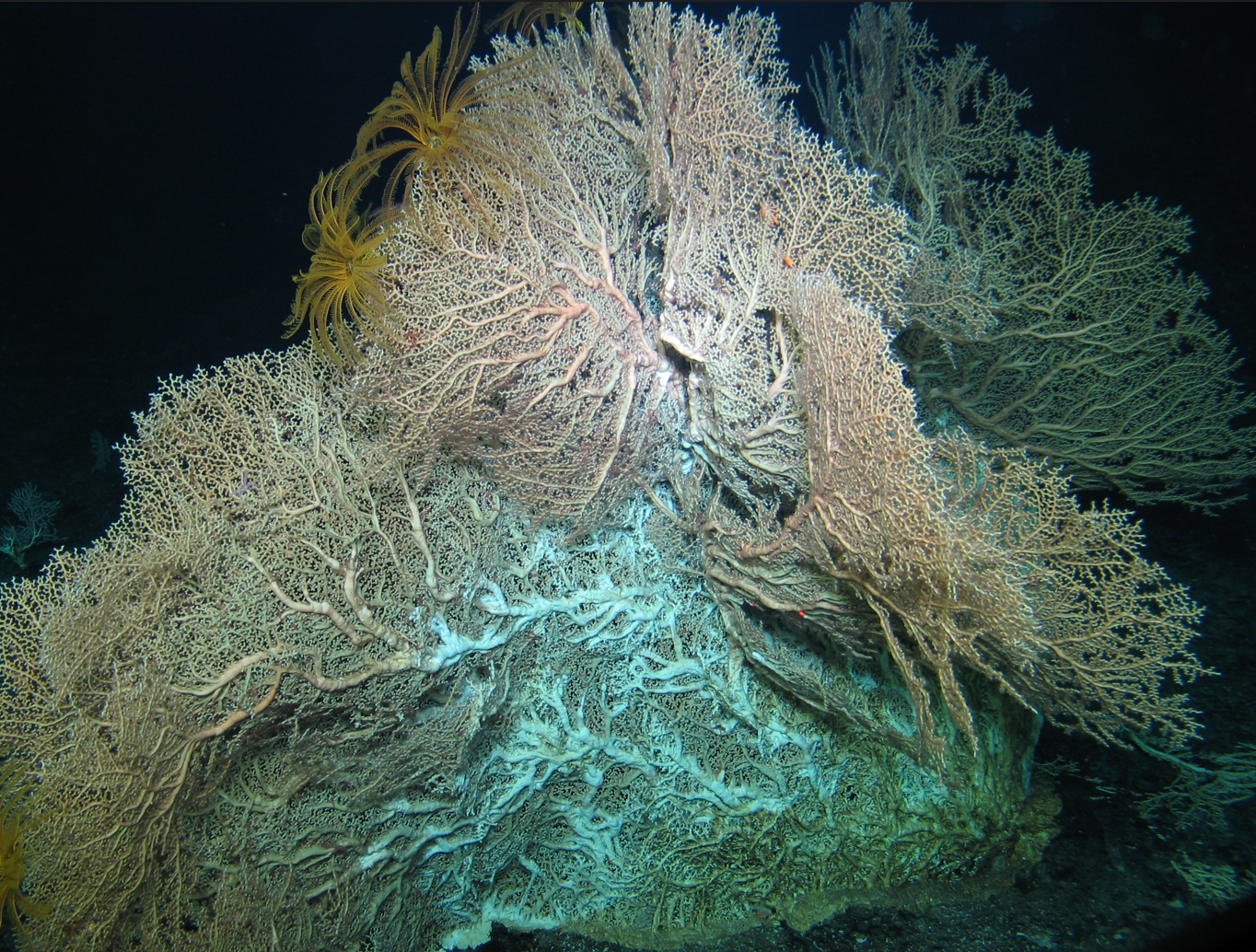 |
| Order: Antipatharia |  |  |
| Family: Cladopathidae | Cladopathidae spp. (branched) indet. | 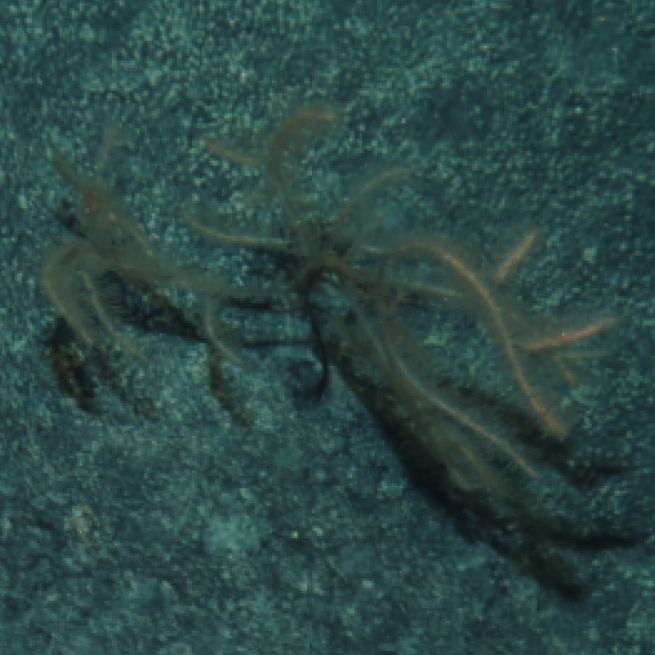 |
| Family: Schizopathidae | *Umbellapathes* sp. indet. | 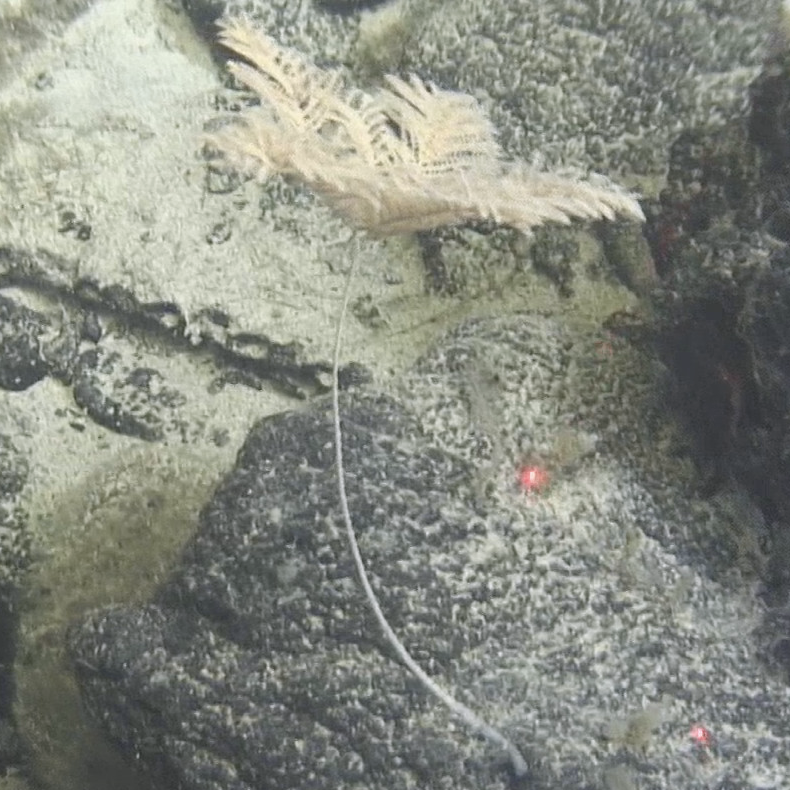 |
|  | *Bathypathes* spp. (fan) indet. | 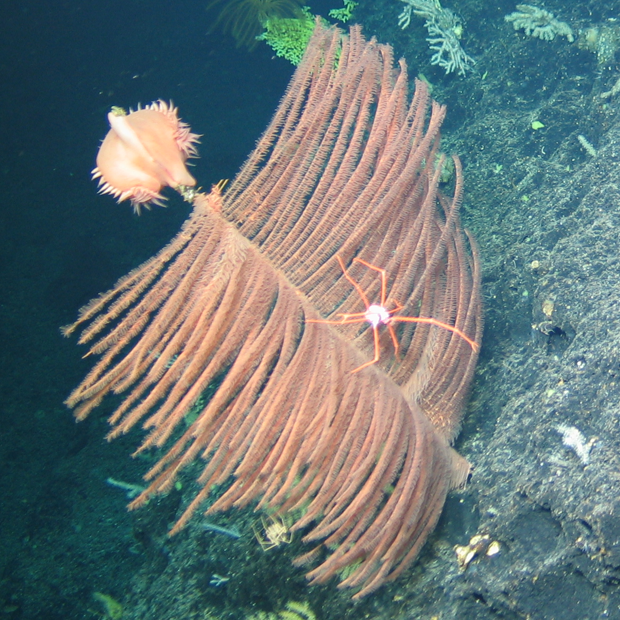 |
| Order: Actiniaria  (Suborder: Enthemonae) |  |  |
| (Superfamily: Metridioidea)  Family: Actinoscyphiidae | Actinoscyphiidae spp. indet. | 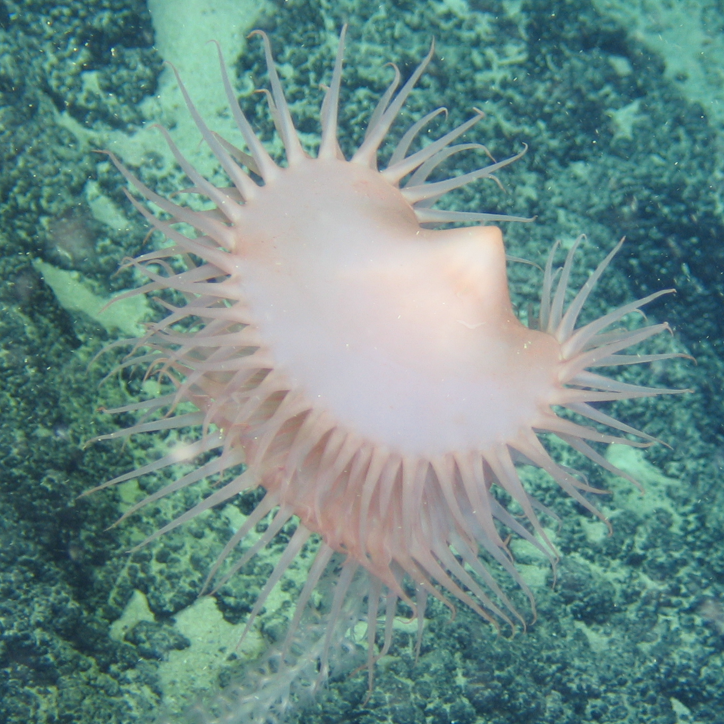 |
| Family: Hormathiidae | Hormathiidae spp. indet. | 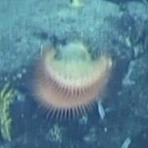 |
| (Superfamily: Actinostoloidea)  Family: Actinostolidae | Actinostolidae spp. indet. | 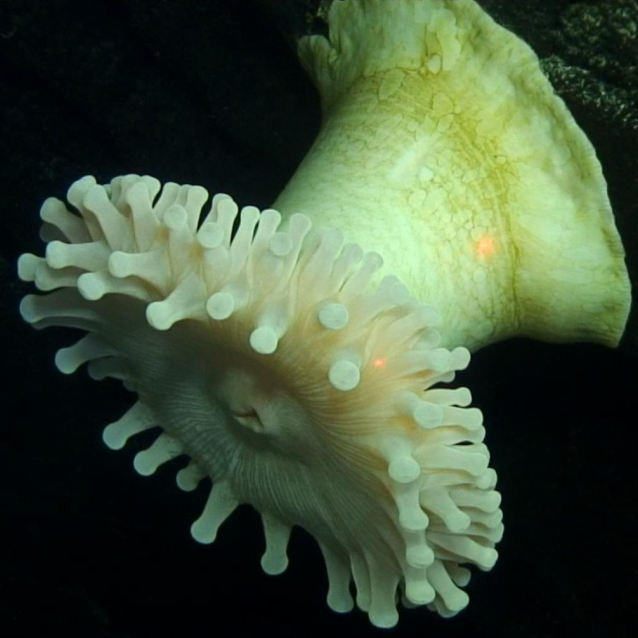 |
| (Suborder: Anenthemonae) |  |  |
| (Superfamily: Actinernoidea)  Family: Actinernidae | Actinernidae sp. indet. | 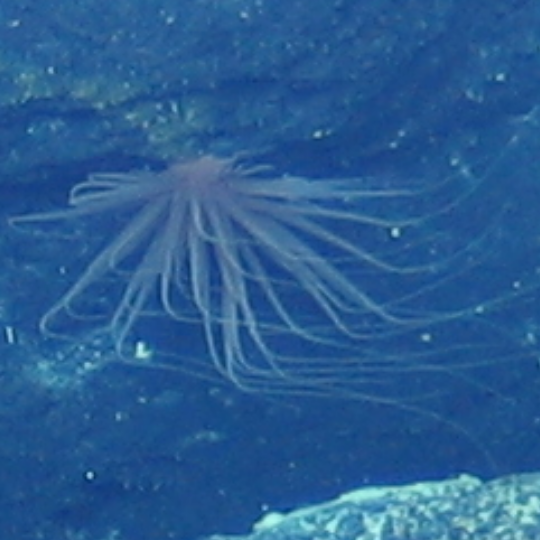 |
| Order: Corallimorpharia |  |  |
| Family: Corallimorphidae | Corallimorphidae spp. indet. | 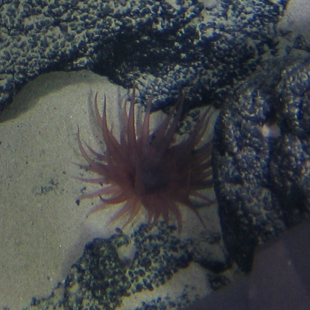 |
| Order: Zoantharia  (Suborder: Macrocnemina) |  |  |
| Family: Epizoanthidae | Epizoanthus spp. indet. | 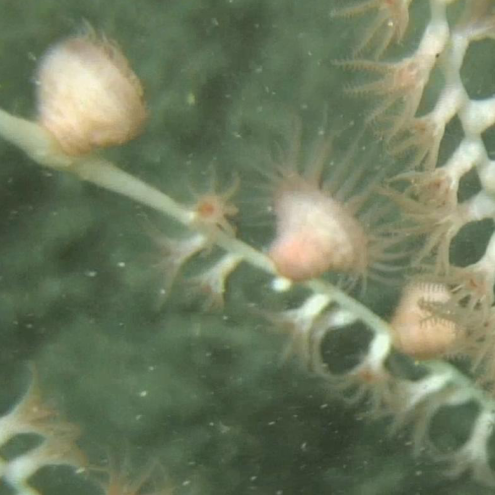 |
| (Subclass: Ceriantharia) |  |  |
| Order: Ceriantharia |  |  |
| Family:Cerianthidae | Cerianthidae sp.indet. | 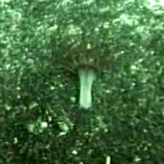 |
| **PHYLUM: PORIFERA** |  |  |
| Class: Hexactinellida  (Subclass: Hexasterophora) |  |  |
| Order: Lyssacinosida |  |  |
| Family: Rossellidae  (Subfamily: Lanuginellinae) | *Caulophacus* spp. indet. | 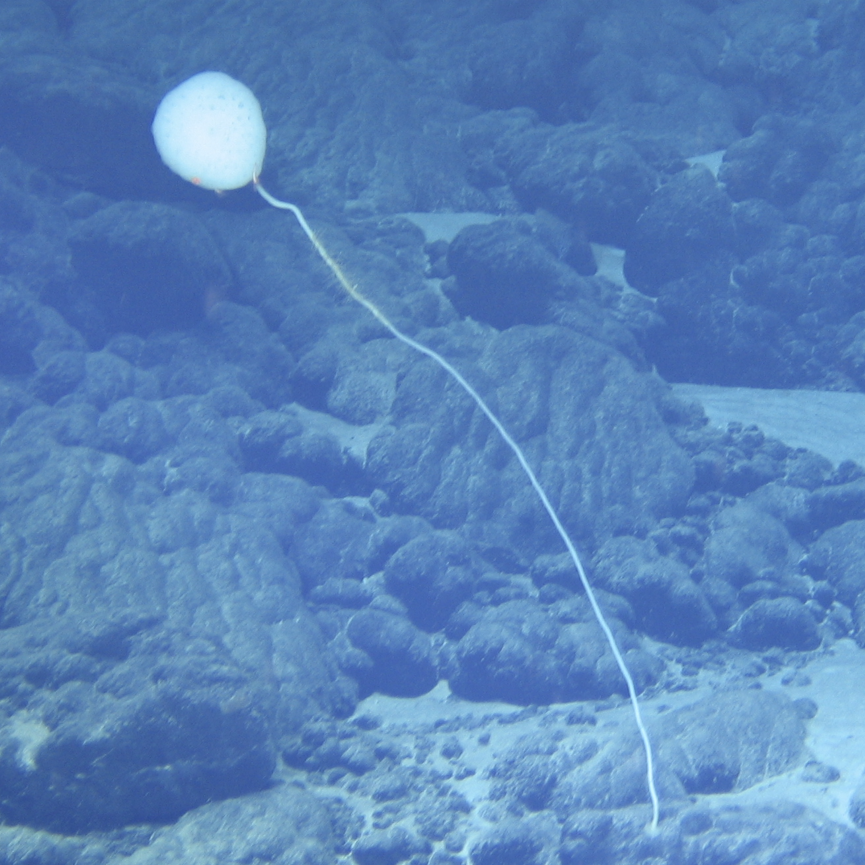 |
| Family: Euplectellidae  (Subfamily: Bolosominae) | *Amphidiscella* spp. indet. | 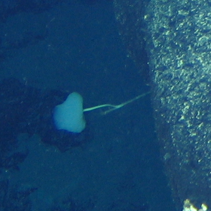 |
|  | *Saccocalyx* *pedunculatus* sp. inc. | 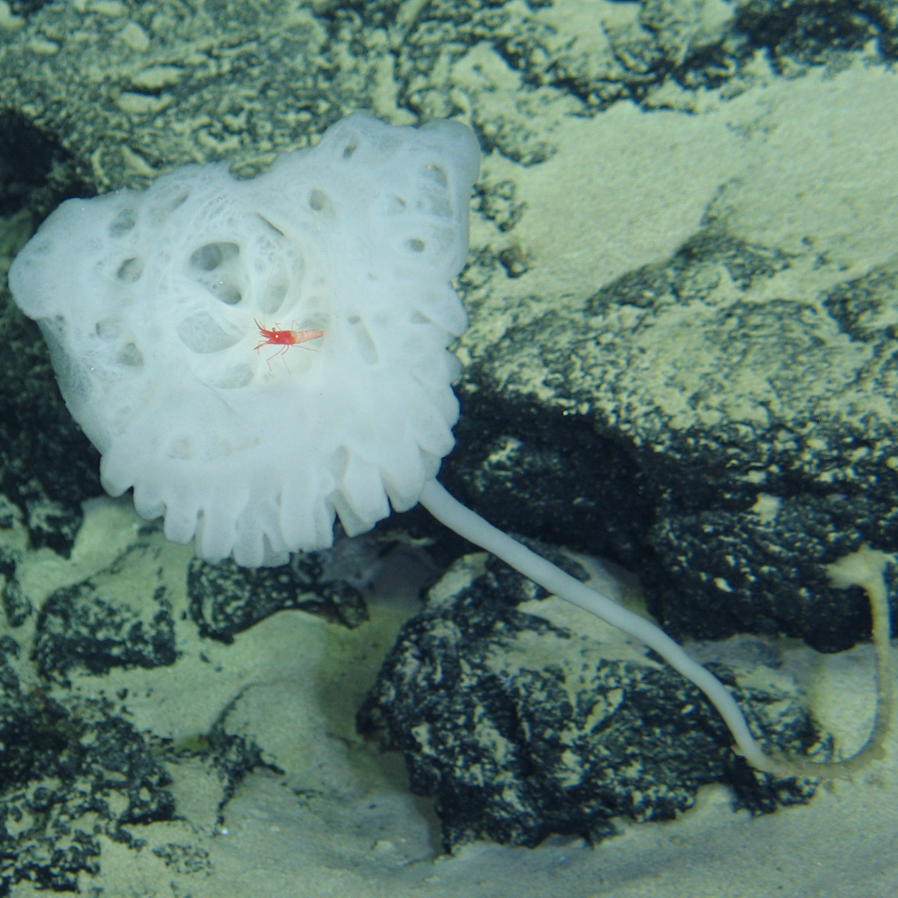 |
| (Subfamily: Corbitellinae) | *Dictyaulus* spp. indet. | 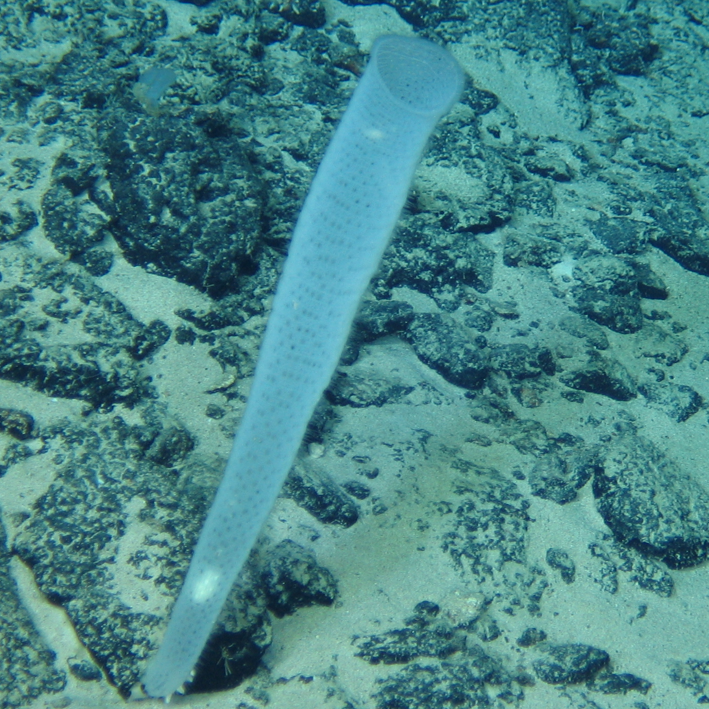 |
|  | Corbitellinae spp. indet. | 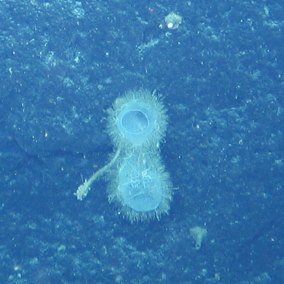 |
|  | *Walteria* *flemmingii* sp. inc. | 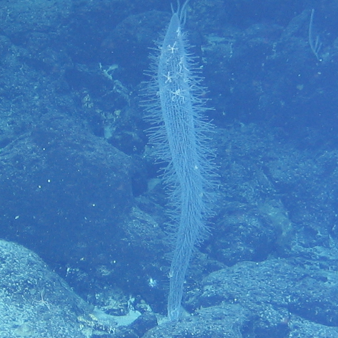 |
|  | *Regadrella okinnoseana* sp. inc. | 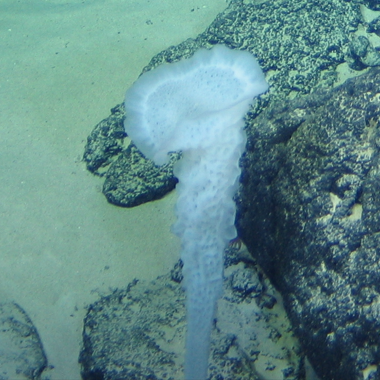 |
| Order: Sceptrulophora |  |  |
| Family: Farreidae | Farreidae spp. indet. | 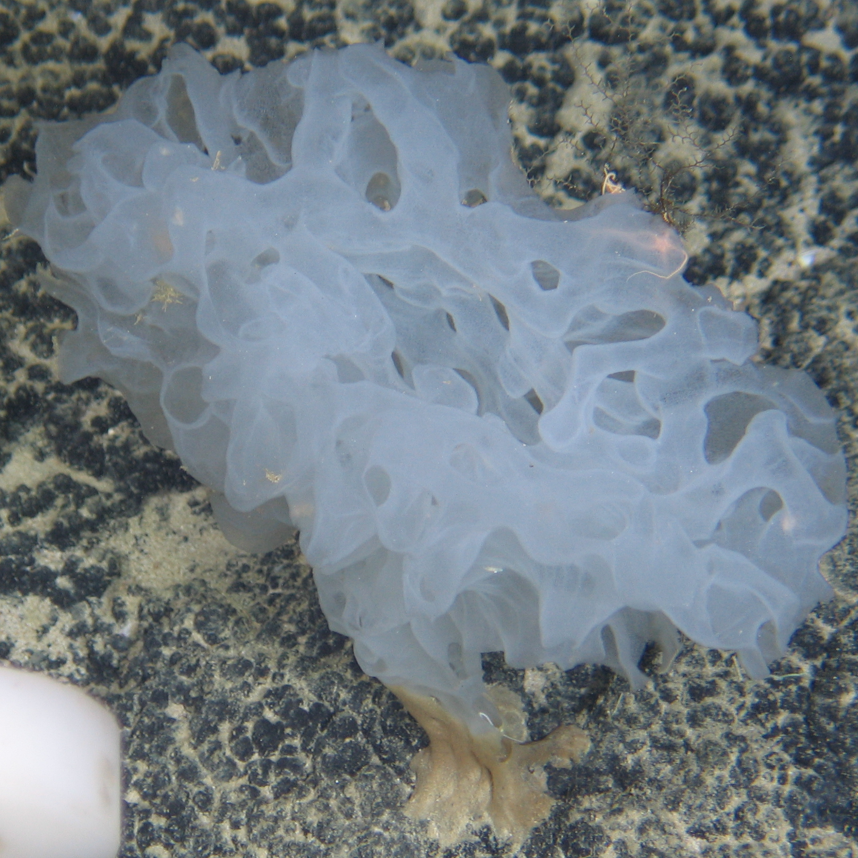 |
| Family: Uncinateridae | *Tretopleura weijica* sp. inc. | 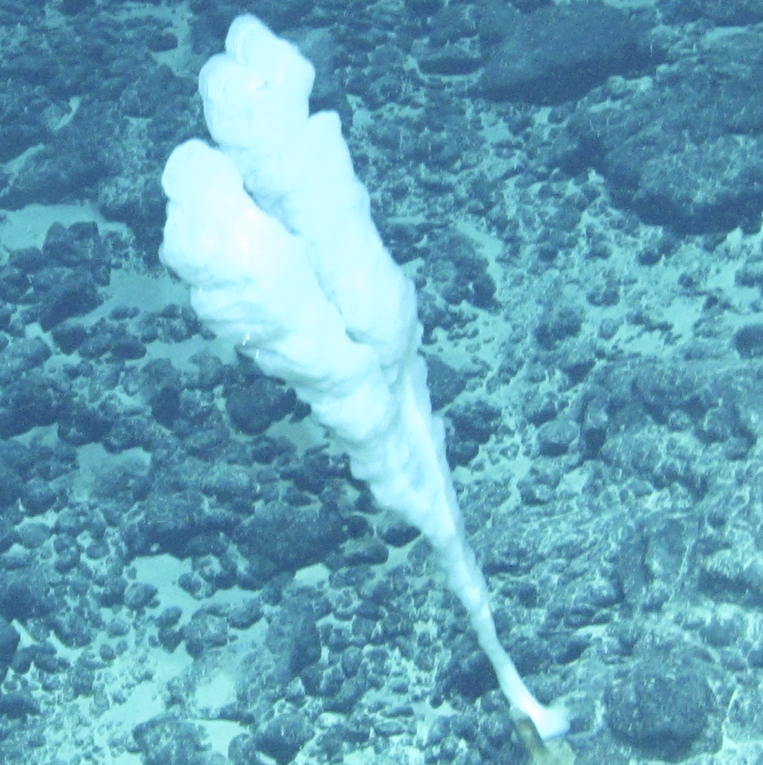 |
| Subclass: Amphidiscophora |  |  |
| Order: Amphidiscosida |  |  |
| Family: Pheronematidae | *Pheronema carpenteri* sp. inc. | 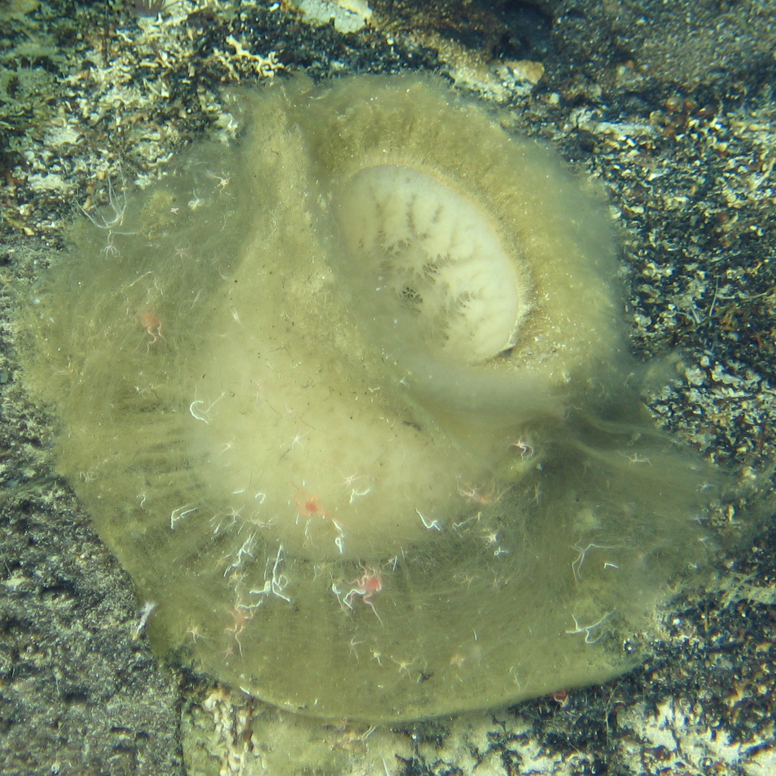 |
|  | *Ijimalophus* spp. indet. | 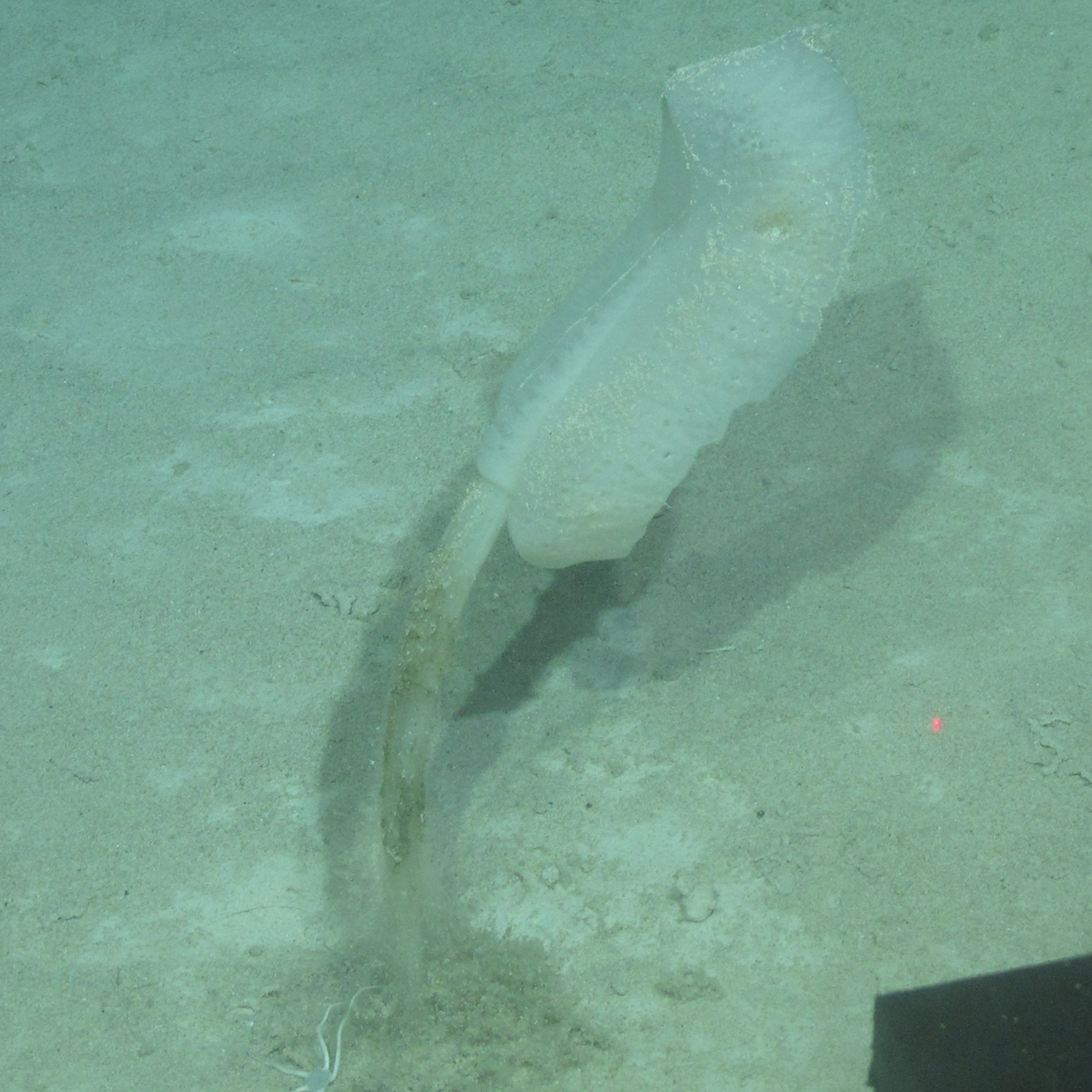 |
|  | *Semperella* spp. indet. | 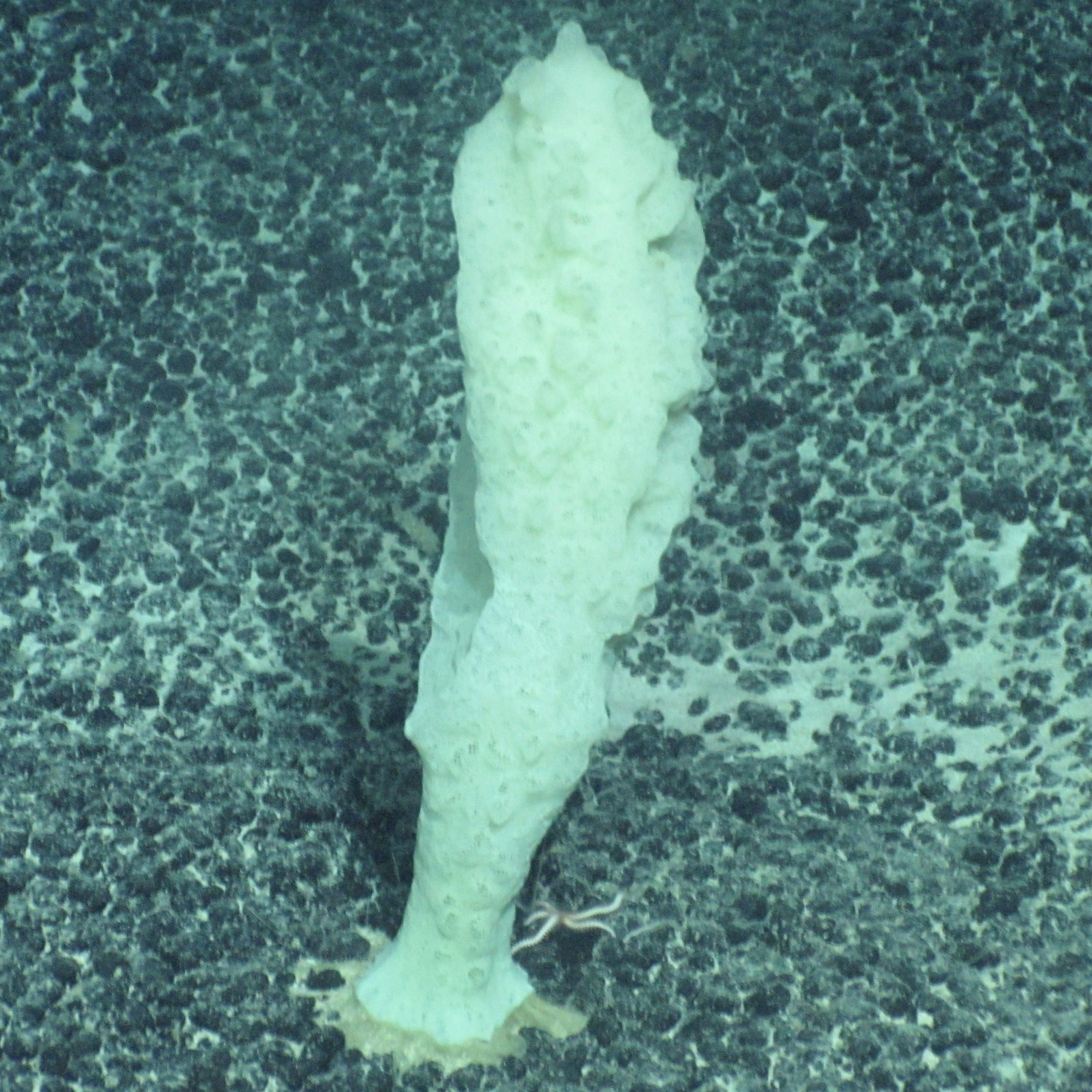 |
|  | *Poliopogon s*pp. indet*.* | 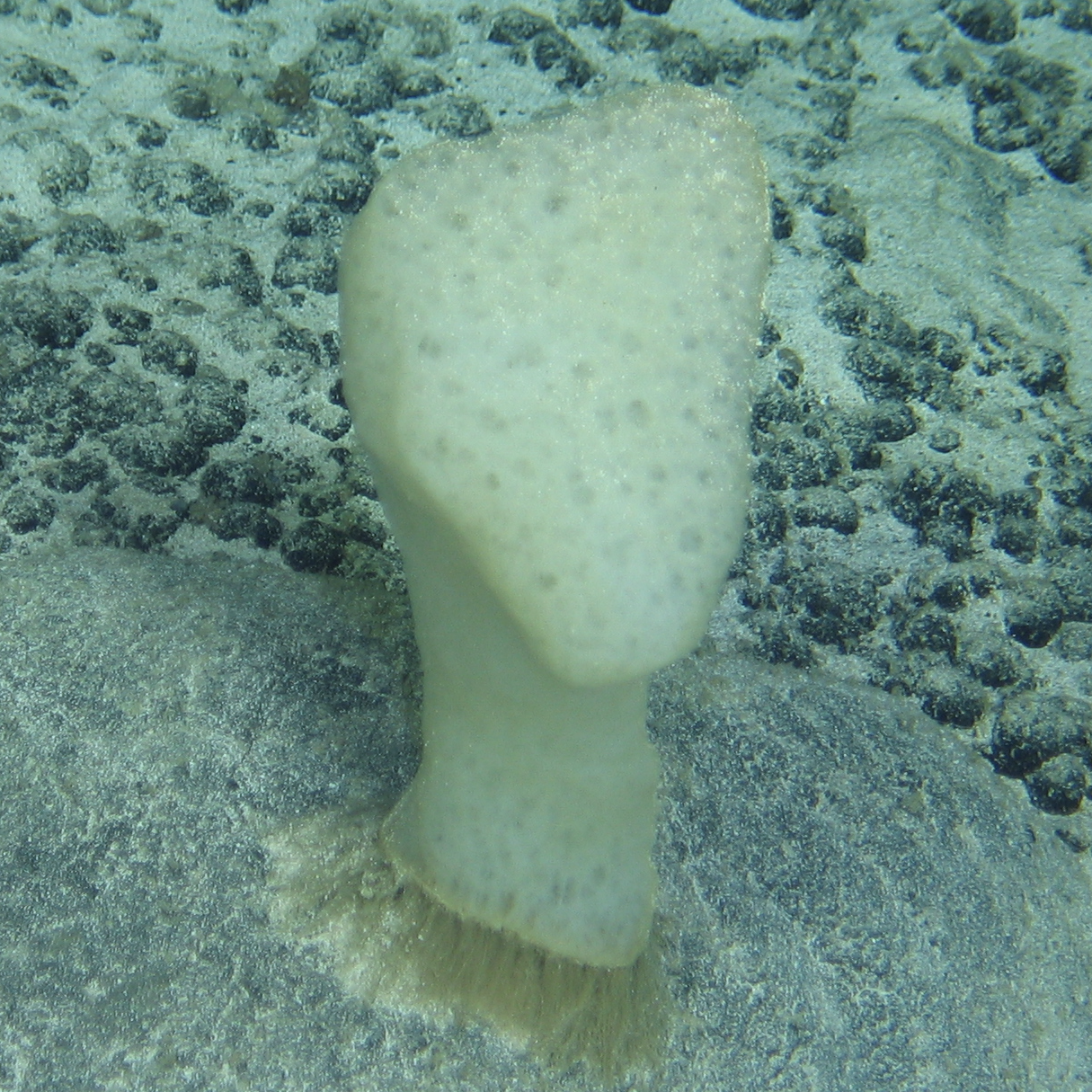 |
|  | *Pheronemoides* sp. indet*.* | 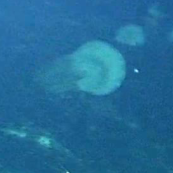 |
| Family: Hyalonematidae | *Hyalonema* spp. indet. | 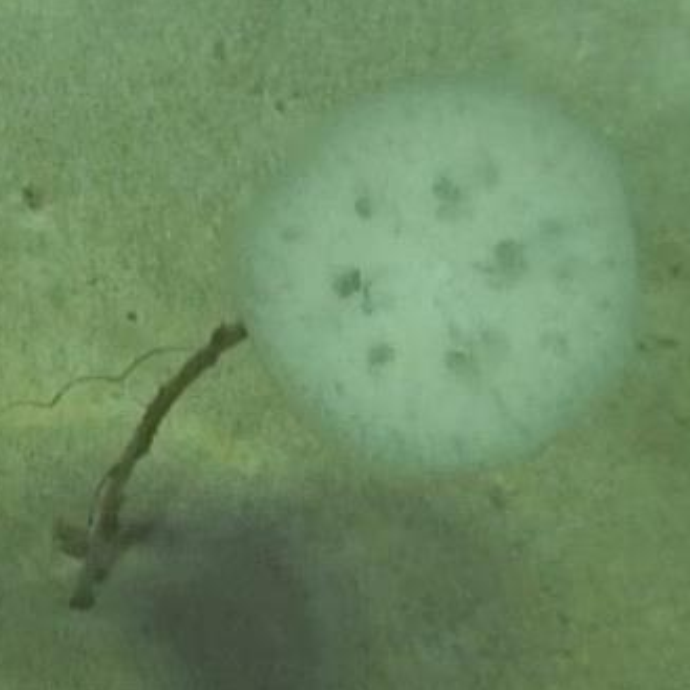 |
|  | Hexactinellida sp. indet. | 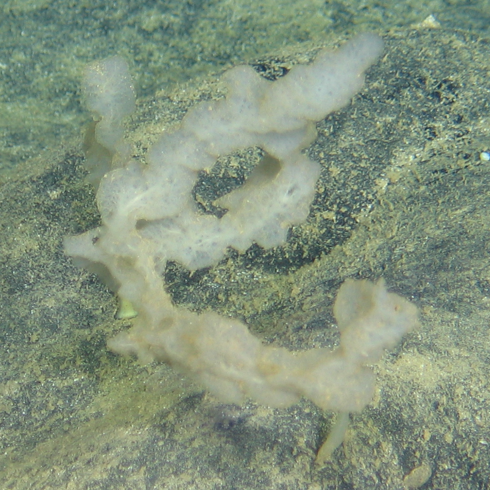 |
| Class: Demospongiae  Subclass: Heteroscleromorpha |  |  |
| Order: Poecilosclerida |  |  |
| Family: Cladorhizidae | *Cladorhiza* spp. indet. | 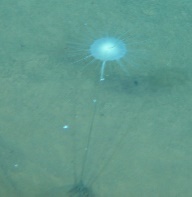 |
| Order: Tetractinellida  Suborder: Astrophorina |  |  |
| Family: Geodiidae | Geodiidae sp. indet. | 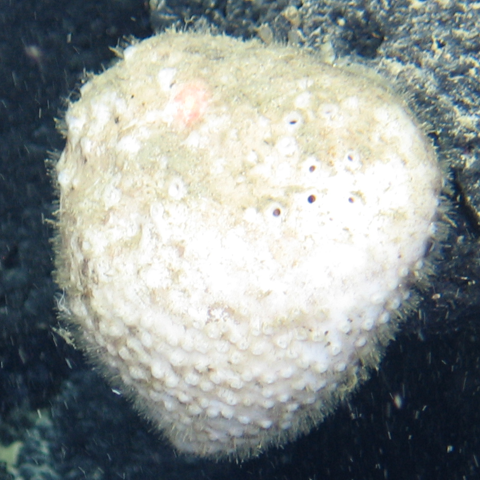 |
| **PHYLUM: ECHINODERMATA** |  |  |
| Class: Crinoidea  Subclass: Articulata |  |  |
| Order: Comatulida |  |  |
| Superfamily: Antedonoidea  Family: Pentametrocrinidae | Pentametrocrinidae spp. indet. | 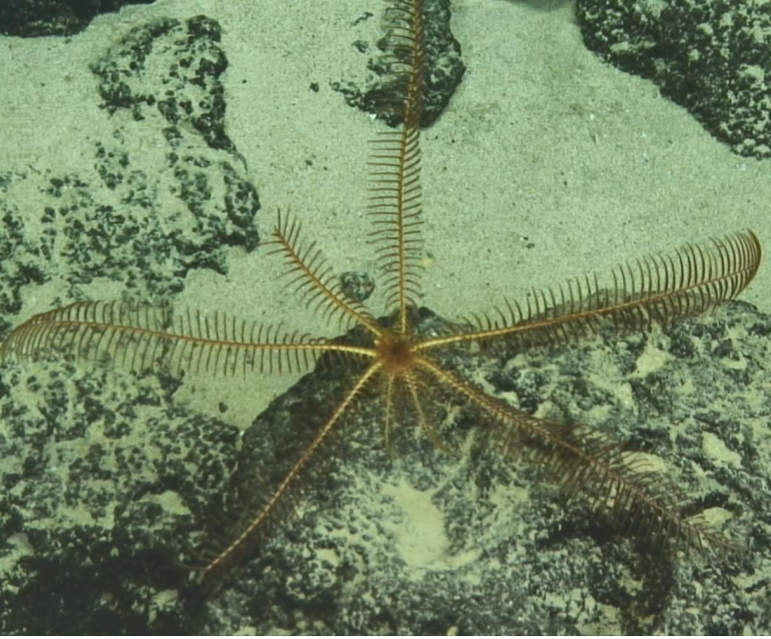 |
| Family: Thalassometridae | *Thalassometra electrae* sp. inc. | 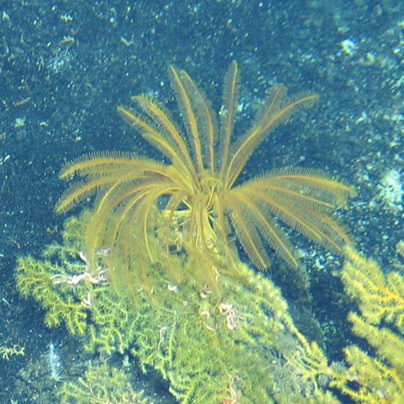 |
| Superfamily: Antedonoidea  Family: Antedonidae | Antedonidae spp. indet. | 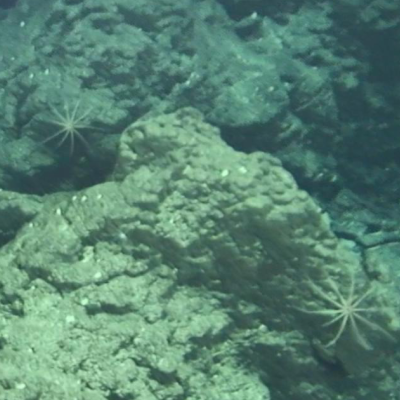 |
| Superfamily: Tropiometroidea  Family: Charitometridae | *Strotometra* spp. indet. | 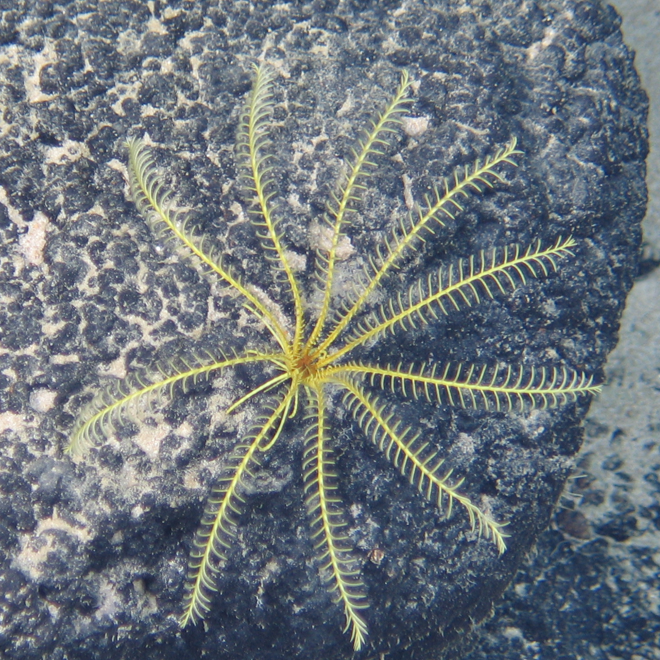 |
| Family: Zenometridae | *Sarametra* spp. indet. | 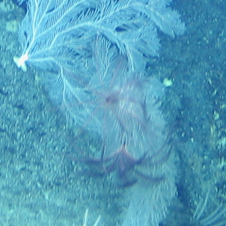 |
| (Suborder: Bourgueticrinina) |  |  |
| Family: Phrynocrinidae  (Subfamily: Phrynocrininae) | *Phrynocrinus nudus* | 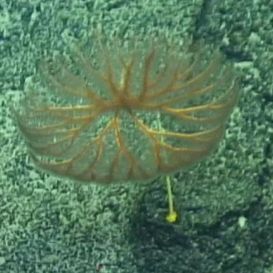 |
| Family: Bathycrinidae  (Subfamily: Bathycrininae) | *Bathycrininae* sp. indet. | 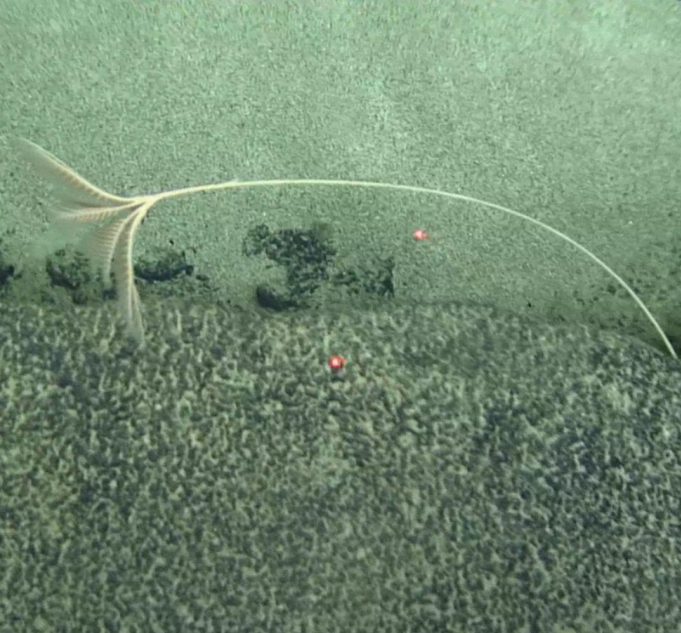 |
| Superfamily: Comatulida incertae sedis  Family: Guillecrinidae | *Guillecrinus* sp. indet. | 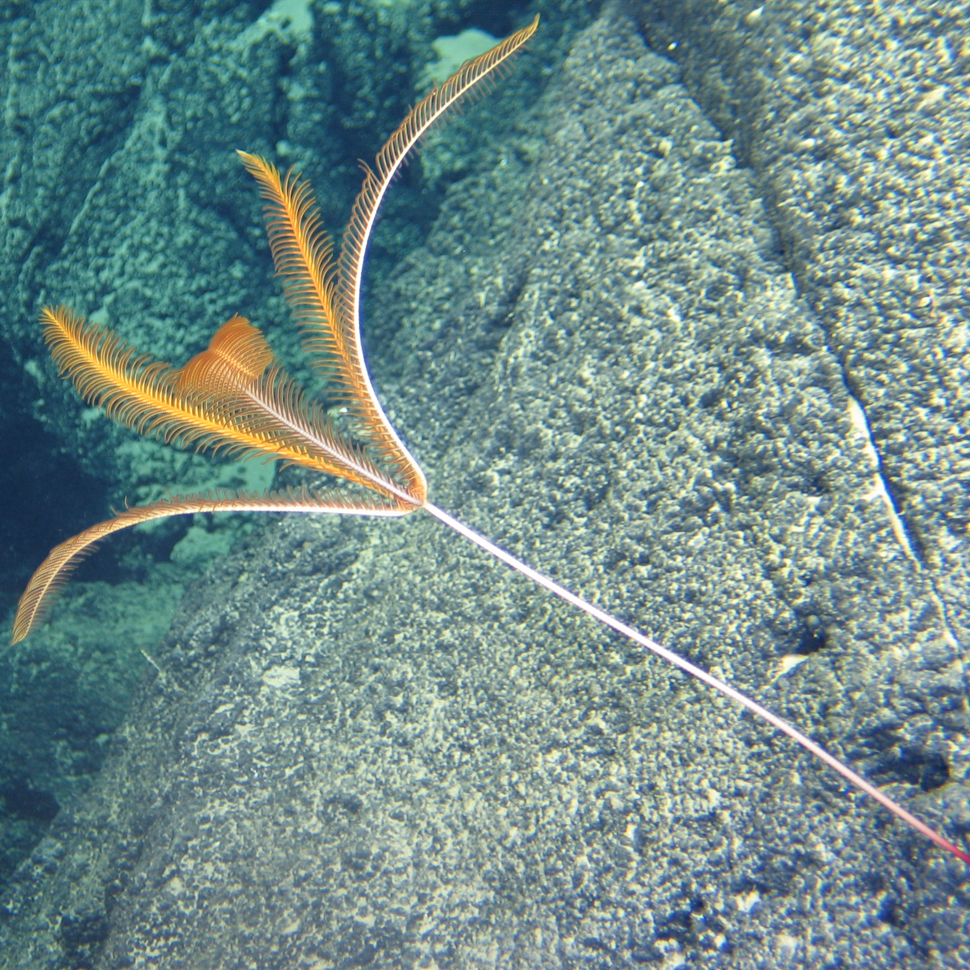 |
| Order: Isocrinida  (Suborder: Isocrinina) |  |  |
| Family: Proisocrinidae | *Proisocrinus ruberrimus* | 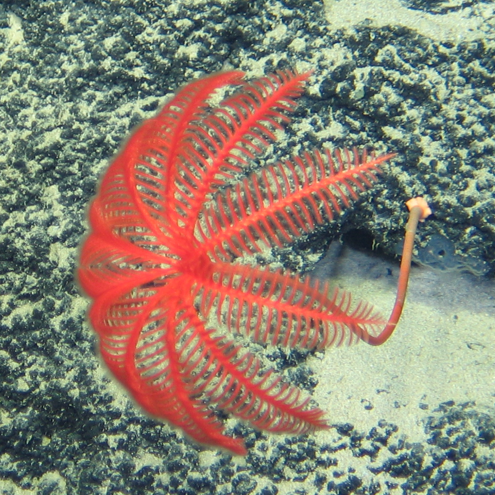 |
| Order: Hyocrinida |  |  |
| Family: Hyocrinidae | Hyocrinidae spp. indet. | 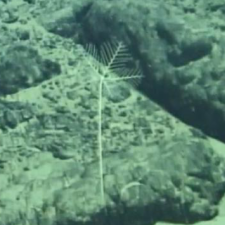 |
| Class: Holothuroidea  Subclass: Actinopoda |  |  |
| Order: Elasipodida |  |  |
| Family: Psychropotidae | *Benthodytes* spp. indet. | 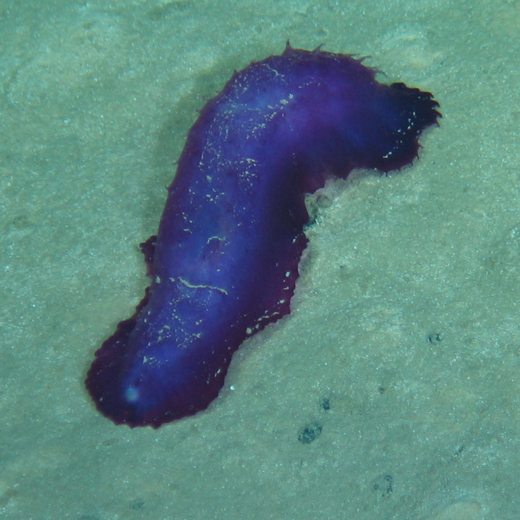 |
| Order: Elasipodida |  |  |
| Family: Pelagothuriidae | *Enypniastes eximia* | 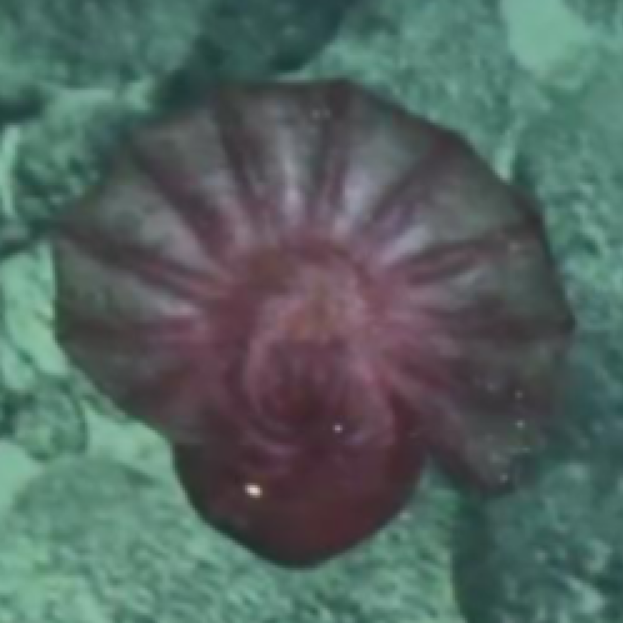 |
| Family: Elpidiidae | *Peniagone* sp. indet. | 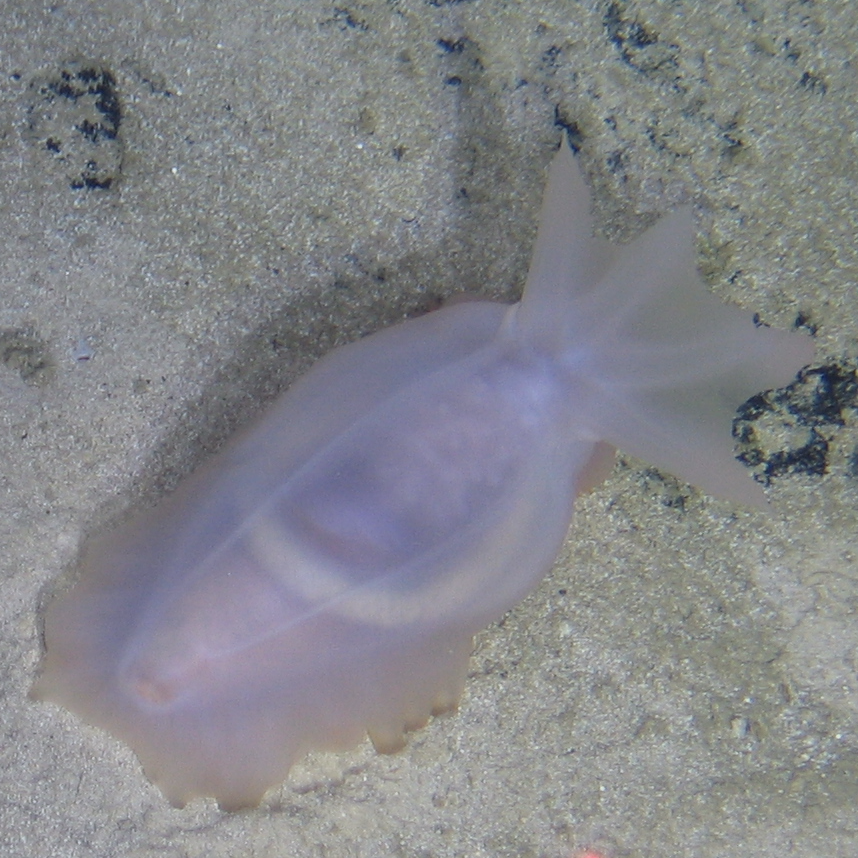 |
| Order: Synallactida |  |  |
| Family: Synallactidae | *Paelopatides* sp. indet. | 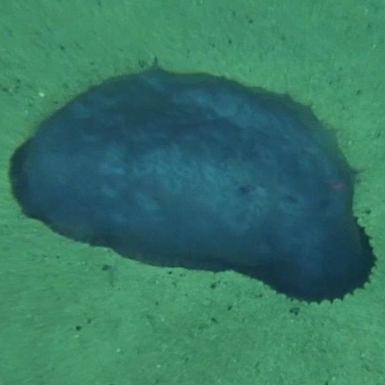 |
| Class: Asteroidea |  |  |
| Superorder: Forcipulatacea  Order: Brisingida |  |  |
| Family: Brisingidae | *Brisinga* spp. indet. | 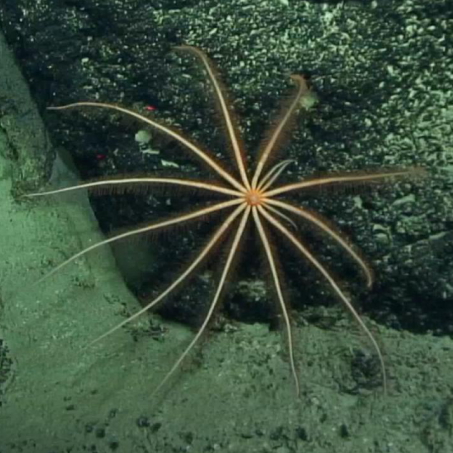 |
|  | *Novodinia* spp. indet. | 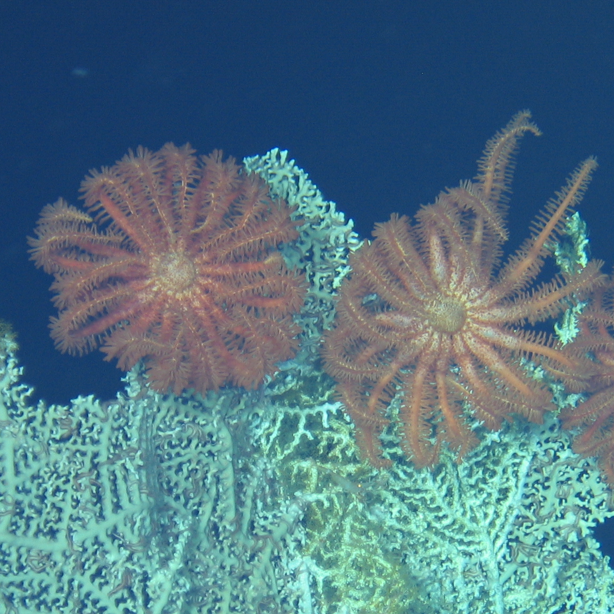 |
|  | *Astrolirus* spp. indet. | 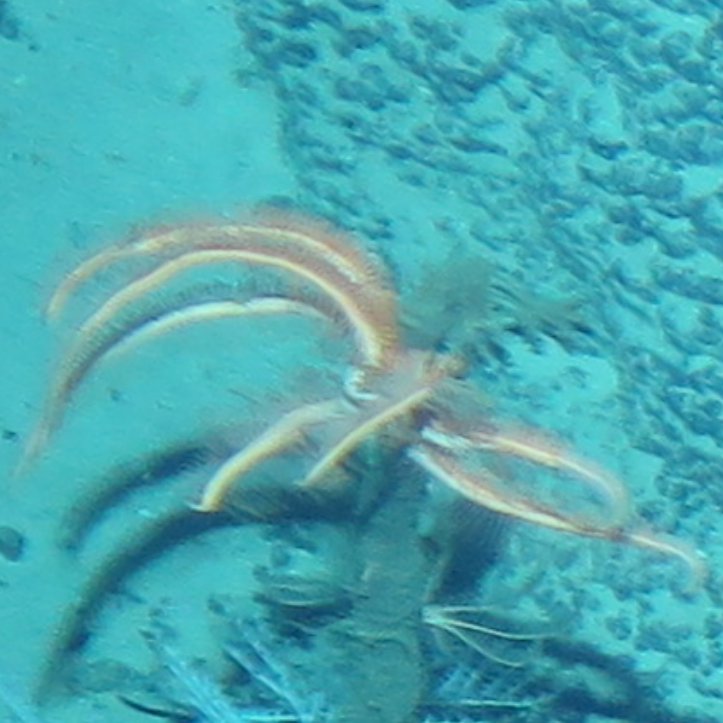 |
| Order: Forcipulatida |  |  |
| Family: Zoroasteridae | *Zoroaster* sp. indet. | 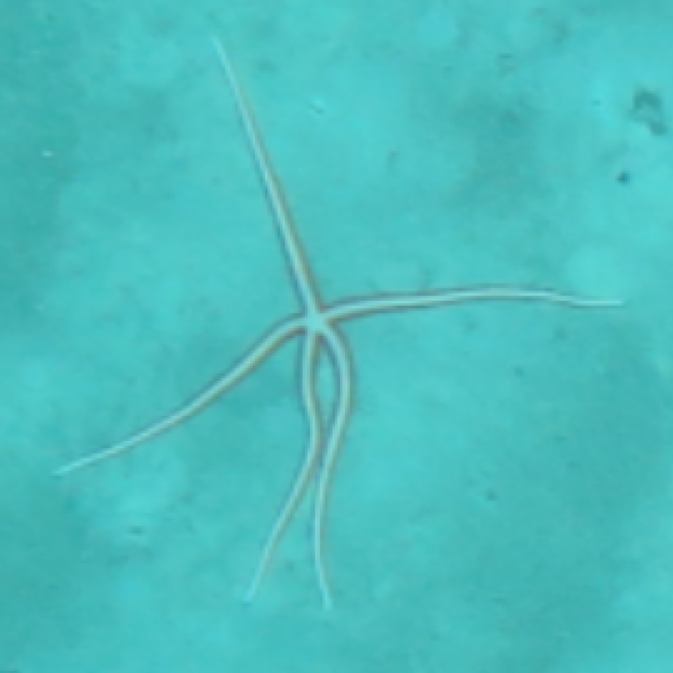 |
| Order: Velatida |  |  |
|  | Velatida sp. indet. | 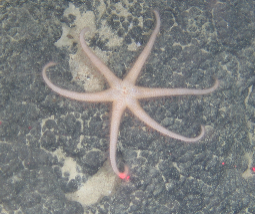 |
| Superorder: Valvatacea  Order: Valvatida |  |  |
| Family: Goniasteridae | *Ceramaster* spp. indet. | 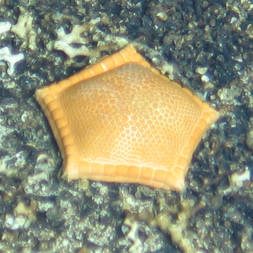 |
| Family: Pterasteridae | *Hymenaster* spp. indet. | 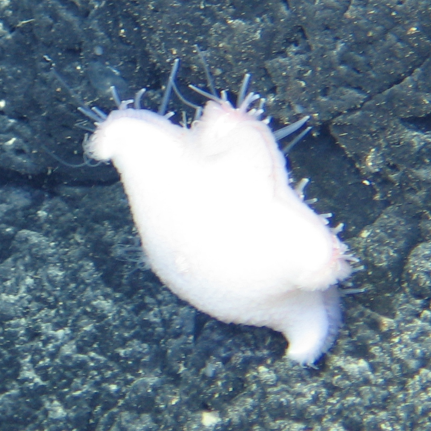 |
|  | *Pteraster* spp. indet. | 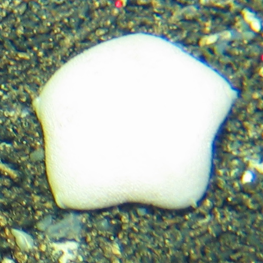 |
| Order: Paxillosida |  |  |
| Family: Benthopectinidae | *Cheiraster* spp. indet. | 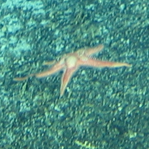 |
| Family: Porcellanasteridae | Porcellanasteridae spp. indet. | 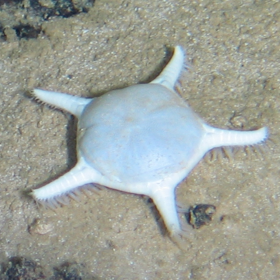 |
| Class: Ophiuroidea  Subclass: Myophiuroida |  |  |
| Superorder: Euryophiurida  Order: Euryalida |  |  |
| Family: Euryalidae | Euryalidae spp. indet. | 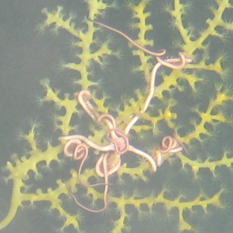 |
| Order: Ophiurida | Ophiurida sp. indet. | 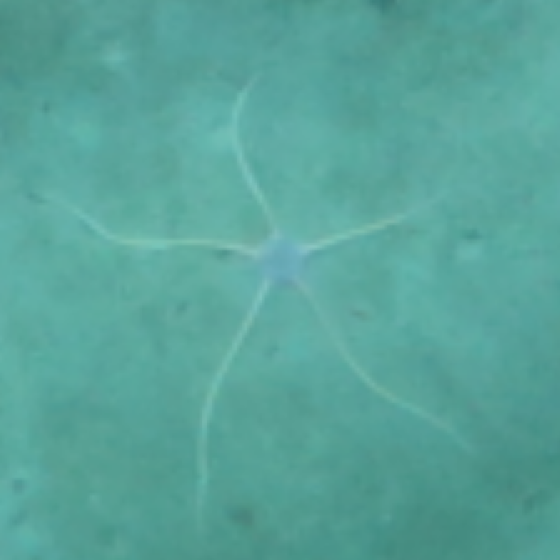 |
| Superorder: Ophintegrida  Order: Ophiacanthida  Suborder: Ophiacanthina |  |  |
| Family: Ophiacanthidae | *Ophioplinthaca defensor* sp. inc. | 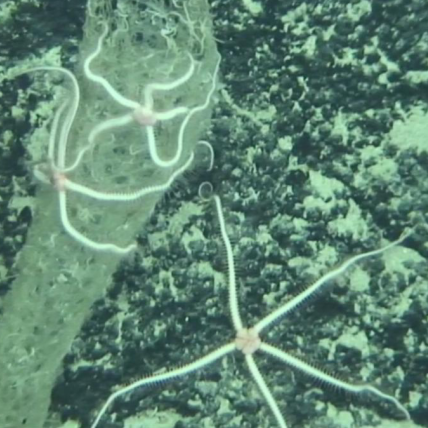 |
| Family: Ophiocamacidae | *Ophiocamax drygalskii* sp. inc. | 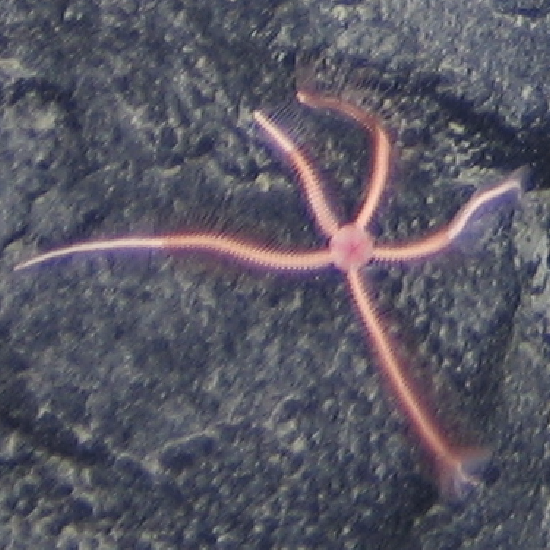 |
| Class: Echinoidea  Subclass: Euechinoidea |  |  |
| Superorder: Echinothuriacea  Order: Aspidodiadematoida |  |  |
| Family: Aspidodiadematidae | *Plesiodiadema* spp. indet. | 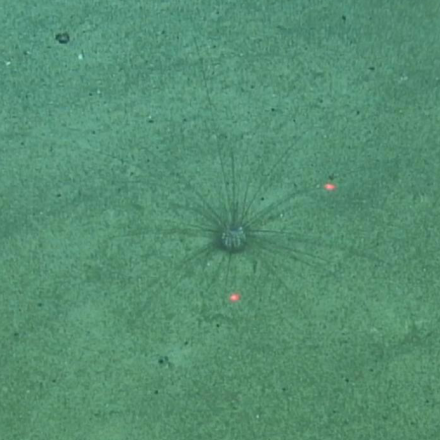 |
| Order: Echinothurioida |  |  |
| Family: Echinothuriidae | Echinothuriidae spp. indet. | 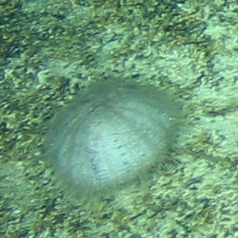 |
| Order: Pedinoida |  |  |
| Family: Pedinidae | *Caenopedina* sp. indet. | 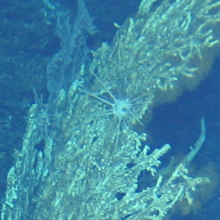 |
| Subclass: Cidaroidea |  |  |
| Order: Cidaroida |  |  |
| Superfamily: Cidaroidea  Family: Cidaridae | *Cidaris* spp. indet. | 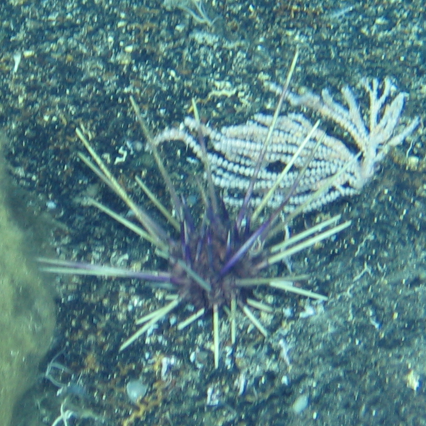 |
| **PHYLUM CHORDATA** |  |  |
| Class: Teleostei |  |  |
| Order: Gadiformes |  |  |
| Family: Macrouridae  (Subfamily: Bythitidae) | *Coryphaenoides* spp. indet. | 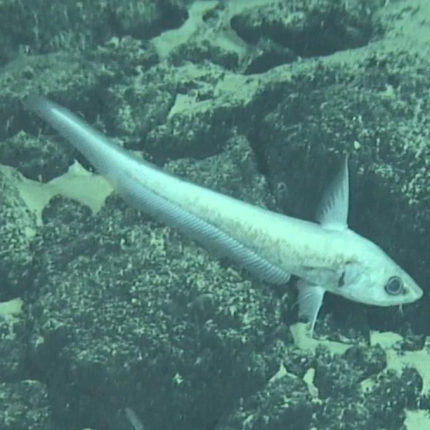 |
| Family: Bythitidae  (Subfamily: Bythitinae) | *Cataetyx* spp. indet. | 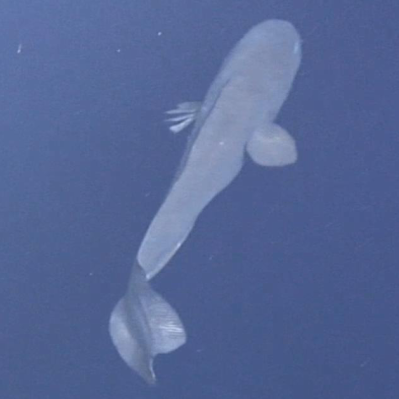 |
| Order: Aulopiformes |  |  |
| Family: Ipnopidae | *Ipnops* spp. indet. | 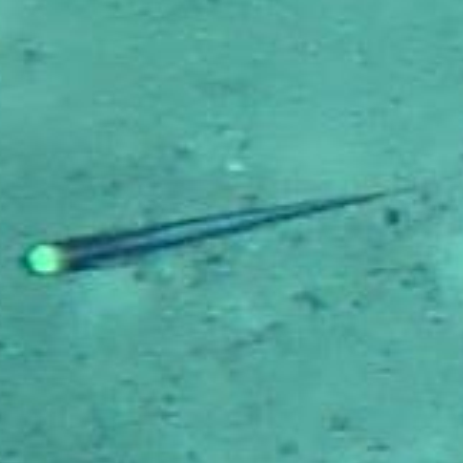 |
| Family: Bathysauroidae | *Bathysaurus* sp. indet. | 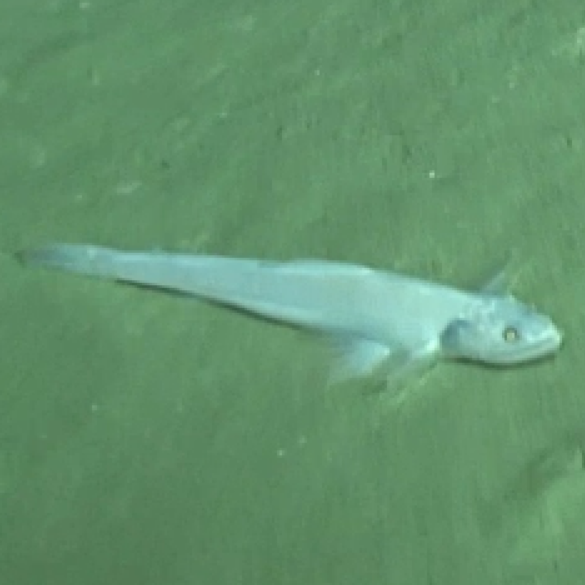 |
| **PHYLUM ARTHROPODA** |  |  |
| Class: Malacostraca  Subclass: Eumalacostraca |  |  |
| Order: Decapoda |  |  |
| Family: Pandalidae | *Heterocarpus* spp.indet. | 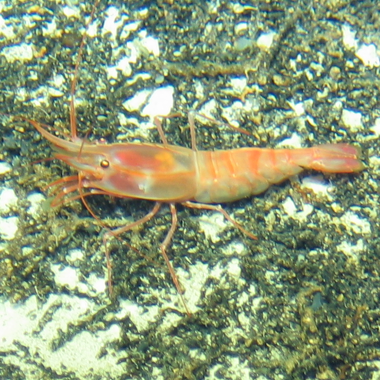 |
| Family: Aristeidae | Aristeidae spp. indet. | 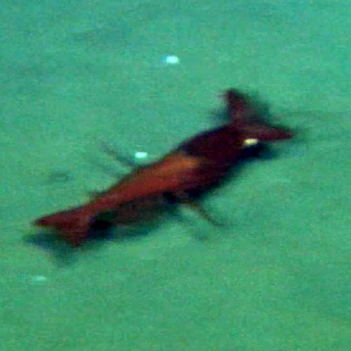 |
| Family: Chirostylidae | Chirostylidae spp. indet. | 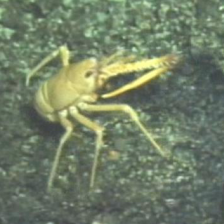 |
| Superfamily: Paguroidea | Paguroidea spp. indet. | 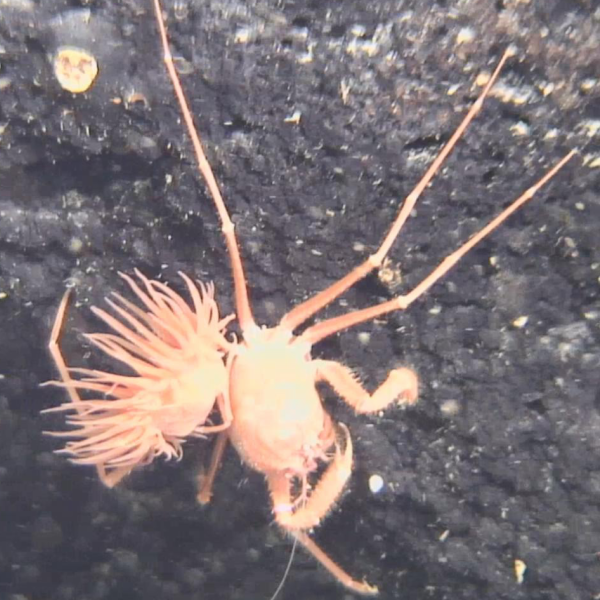 |

## Supplementary Table S3.

Medoids of Species (unit: %) for each cluster is calculated by noise clustering to demonstrate the relative importance of each morphospecies within each cluster. Only the morphospecies with medoids greater than 5% in at least one of the clusters were shown. The values of medoids greater than 10% were shown in bold.

| Names of morphospecies | Cluster | | | | | |
| --- | --- | --- | --- | --- | --- | --- |
|  | M1 | M2 | M3 | M4 | M5 | M6 |
| *Iridogorgia* spp. indet. | 0.3 | 5.6 | 0.6 | 0.4 | 3.8 | 0.4 |
| Plexauridae spp. indet. | **11.7** | 6.0 | 1.1 | 1.5 | 5.9 | 1.5 |
| Primnoidae spp. 1 (sparse) indet. | **58.2** | 5.4 | 0.9 | 1.3 | 6.2 | 1.4 |
| Primnoidae spp. 2 (dense) indet. | **54.8** | 1.6 | 0.7 | 1.3 | 4.0 | 1.4 |
| *Callogorgia* spp. indet. | 6.2 | 0.1 | 0.1 | 0.1 | 0.2 | 0.1 |
| *Paracalyptrophora* spp. indet. | **13.8** | 0.5 | 0.4 | 0.8 | 1.0 | 0.8 |
| *Lepidisis* spp. indet. | 1.6 | **10.1** | 5.0 | 6.5 | **61.8** | 2.1 |
| *Umbellula* spp. indet. | 0.1 | 4.7 | 2.9 | 0.1 | 0.2 | **80.1** |
| *Caulophacus* spp. indet. | 2.1 | 1.6 | 1.2 | 5.9 | **12.6** | 1.1 |
| Amphidiscella spp. indet. | 1.1 | 1.4 | 3.6 | 1.8 | 5.4 | 1.8 |
| *Dictyaulus* spp. indet. | 1.3 | 8.2 | 5.4 | 1.4 | 7.5 | 1.2 |
| *Walteria* *flemmingii* sp. inc. | 5.9 | 0.0 | 0.0 | 0.1 | 0.1 | 0.1 |
| *Pheronema carpenteri* sp. inc. | 8.9 | 0.5 | 0.2 | 0.4 | 0.8 | 0.5 |
| Cladorhiza spp. indet. | 0.1 | 0.1 | 0.1 | 0.3 | 0.3 | 6.5 |
| *Hyalonema* spp. indet. | 0.3 | 0.4 | 0.4 | 4.2 | 5.3 | 0.9 |
| Pentametrocrinidae spp. indet. | 1.4 | **78.2** | 1.9 | 1.3 | 7.3 | 1.3 |
| *Strotometra* spp. indet. | 0.3 | **14.2** | 0.5 | 0.6 | 5.2 | 0.6 |
| *Benthodytes* spp. indet. | 0.3 | 1.4 | 0.8 | **77.5** | 3.7 | 0.7 |
| *Brisinga* spp. indet. | 0.8 | 9.7 | **86.3** | 7.3 | **10.7** | 1.1 |
| Ophioplinthaca spp. indet. | 2.1 | 5.4 | 0.3 | 0.6 | 0.8 | 0.6 |
| Echinothuriidae spp. indet. | 0.8 | 1.6 | 0.6 | 1.1 | 1.3 | 6.3 |

## Supplementary Figure S1.

Plot of Pearson correlation analysis of environmental variables


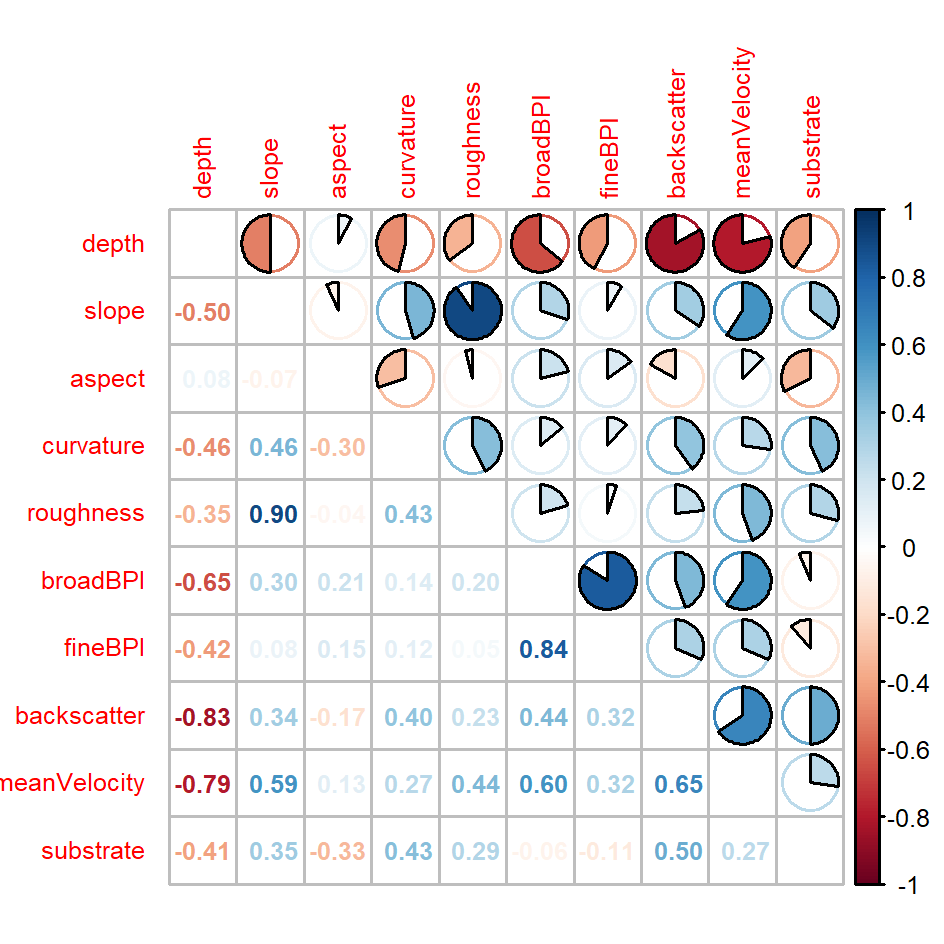


## Supplementary Figure S2.

Water mass properties at the station CTD18 (134.57E, 12.61N). Temperature and Salinity conditions through a vertical profile from the surface to 3300 m.


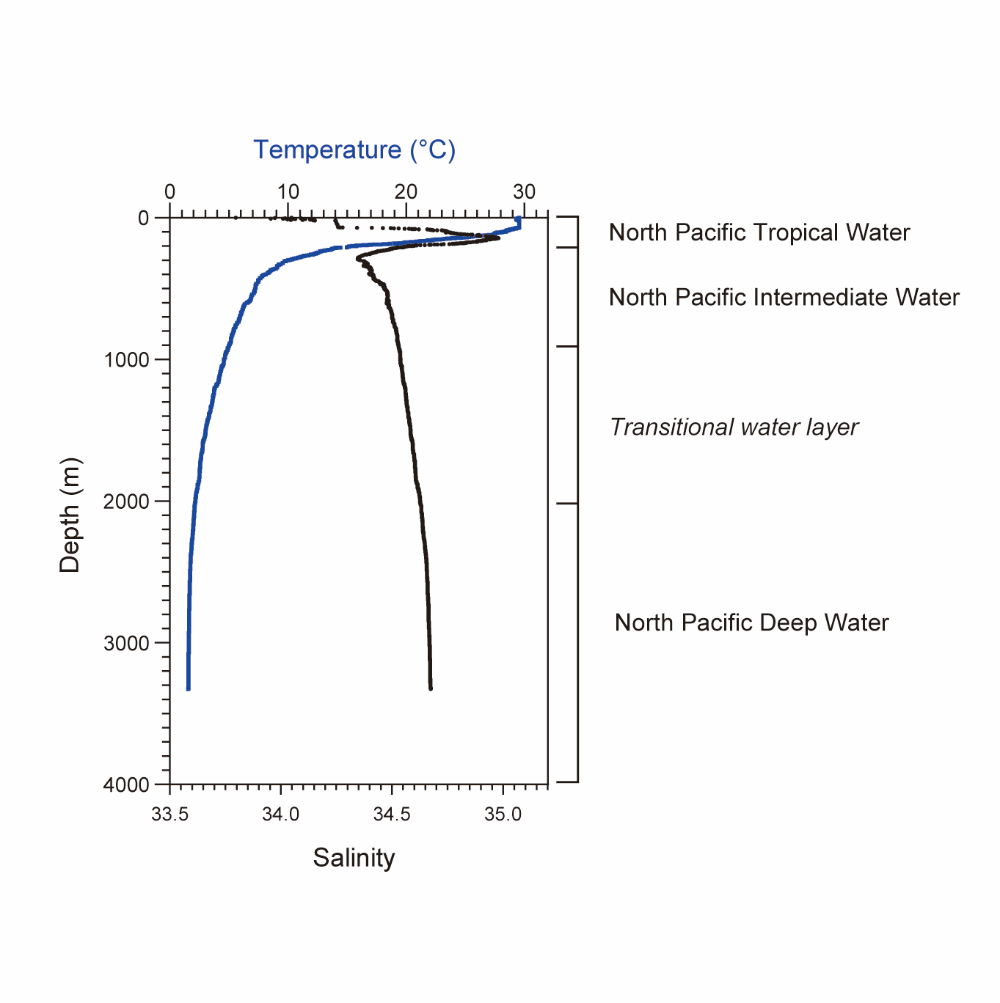

Supplement: Supplementary file 1 — Appendix S1. [file ECE3-14-e70427-s001.docx]
